# Ubiquitination-Related Gene Signature, Nomogram and Immune Features for Prognostic Prediction in Patients with Head and Neck Squamous Cell Carcinoma

**DOI:** 10.3390/genes15070880

**Published:** 2024-07-04

**Authors:** Huiwen Yang, Liuqing Zhou, Mengwen Shi, Jintao Yu, Yi Xie, Yu Sun

**Affiliations:** 1 Department of Otorhinolaryngology, Union Hospital, Tongji Medical College, Huazhong University of Science and Technology, Wuhan 430022, China; xyhw@hust.edu.cn (H.Y.); 2013xh0823@hust.edu.cn (L.Z.); m202175945@hust.edu.cn (M.S.); jintaoyu@hust.edu.cn (J.Y.); 2 Intelligent Medical Laboratory, Union Hospital, Tongji Medical College, Huazhong University of Science and Technology, Wuhan 430022, China; 3 Hubei Province Key Laboratory of Oral and Maxillofacial Development and Regeneration, Wuhan 430022, China; 4 Institute of Otorhinolaryngology, Union Hospital, Tongji Medical College, Huazhong University of Science and Technology, Wuhan 430022, China

**Keywords:** ubiquitination-related gene, head and neck squamous cell carcinoma, TCGA, bioinformatics analysis, prognosis

## Abstract

The objective of this research was to create a prognostic model focused on genes related to ubiquitination (UbRGs) for evaluating their clinical significance in head and neck squamous cell carcinoma (HNSCC) patients. The transcriptome expression data of UbRGs were obtained from The Cancer Genome Atlas (TCGA) database, and weighted gene co-expression network analysis (WGCNA) was used to identify specific UbRGs within survival-related hub modules. A multi-gene signature was formulated using LASSO Cox regression analysis. Furthermore, various analyses, including time-related receiver operating characteristics (ROCs), Kaplan–Meier, Cox regression, nomogram prediction, gene set enrichment, co-expression, immune, tumor mutation burden (TMB), and drug sensitivity, were conducted. Ultimately, a prognostic signature consisting of 11 gene pairs for HNSCC was established. The Kaplan–Meier curves indicated significantly improved overall survival (OS) in the low-risk group compared to the high-risk group (*p* < 0.001), suggesting its potential as an independent and dependable prognostic factor. Additionally, a nomogram with AUC values of 0.744, 0.852, and 0.861 at 1-, 3-, and 5-year intervals was developed. Infiltration of M2 macrophages was higher in the high-risk group, and the TMB was notably elevated compared to the low-risk group. Several chemotherapy drugs targeting UbRGs were recommended for low-risk and high-risk patients, respectively. The prognostic signature derived from UbRGs can effectively predict prognosis and provide new personalized therapeutic targets for HNSCC.

## 1. Introduction

Head and neck cancer is a lethal malignancy, ranking among the top ten most prevalent tumors in both men and women worldwide. The year 2020 witnessed a number of new cases at 931,922 and 467,125 fatalities [1,2]. In the United States alone, there were approximately 136,550 newly diagnosed cases and a death toll of around 35,000 [3]. Head and neck squamous cell carcinoma (HNSCC) stands as the predominant type within this category, constituting more than 90% of all head and neck cancers [4]. Recent years have seen remarkable progress in targeted therapy alongside the emergence of immunotherapy as an innovative treatment modality [5,6,7]. Although targeted immunotherapy has shown enhanced patient survival rates in HNSCC, the proportion of patients who exhibit a lasting response to these treatments is limited to only 15–20% [8]. Even though patients with locoregionally advanced HNSCC are treated with surgery, radiotherapy, chemotherapy, immunotherapy, or combination therapy, the five-year overall survival (OS) rate remains suboptimal [9]. Therefore, it is essential to discover dependable diagnostic indicators and to pinpoint new intervention targets and prognosis factors.

Ubiquitination, a reversible and dynamic post-translational modification, is a crucial regulator of cellular protein degradation pathways [10,11]. The ubiquitination pathway entails the conjugation of ubiquitin molecules to the target protein, followed by its recognition and subsequent degradation mediated by proteasomes [12]. In human cells, more than 80% of protein is degraded through the ubiquitination enzyme pathway [13]. Ubiquitination not only affects the protein localization, metabolism, function, regulation and degradation [14], but it also regulates various cellular processes such as cell cycle, proliferation, apoptosis [15,16], differentiation, inflammation, and immune response [17,18]. The organism’s health cannot be separated from the harmonious equilibrium between the ubiquitination and deubiquitination of intracellular proteins, which dynamically adjusts in response to physiological demands. A growing body of reports suggests that abnormal control of the ubiquitination pathway could lead to various diseases, including malignancies and autoimmune disorders [19,20,21,22]. For instance, the E3 ubiquitin ligase HRD1 promotes lung cancer through ubiquitination of the Sirtuin 2 protein [23]. The elevated expression of Cullin1, a pivotal scaffold protein in the ubiquitin E3 ligase complex, was significantly correlated with inferior overall survival and lymph node metastasis in gastric cancer [24]. Therefore, investigating the dysregulated ubiquitin system as a promising therapeutic target for these diseases holds significant scientific and clinical implications. Precision-targeted ubiquitination and degradation of oncogene proteins offer bright prospects for drug development in cancer. In addition, molecular subtyping and risk stratification tools could be developed for prognostic prediction using ubiquitination-related genes (UbRGs) [25]. However, few studies have been conducted to determine whether there is a correlation between ubiquitination and the outcome assessment of HNSCC.

The ubiquitin proteasome system is essential for protein degradation and is associated with oncogenesis. Therefore, our aim was to investigate the involvement of all known UbRGs in HNSCC. Utilizing the TCGA dataset, we screened for UbRGs associated with outcomes in HNSCC patients using various statistical strategies. The main contributions of this work are as follows: (1) we established a novel and reliable risk score model based on these screened UbRGs, which can be employed to predict the prognosis of HNSCC patients; (2) based on this risk model, we developed a nomogram for individualized prognosis assessment to assist clinicians in making prognostic judgments for individual patients; (3) additionally, we characterized the immune landscape of HNSCC patients in relation to the prognostic risk model, providing valuable references for future studies on HNSCC immunization. In the future, personalized precision treatment and prognosis assessment will be paramount in cancer patient care, and we believe that research on UbRG-related HNSCC patient prognoses is meaningful.

## 2. Materials and Methods

### 2.1. Collection of UbRGs and Sample Data

In this research, we downloaded a total of 1058 UbRGs from the GeneCards database (https://www.genecards.org/, accessed on 15 January 2024) based on their relevance score being ≥ 5. The Cancer Genome Atlas (TCGA) database (https://portal.gdc.cancer.gov/, accessed on 15 January 2024) was employed for the acquisition of transcriptome expression profiles, clinical characteristic data, and somatic mutation data from patients diagnosed with HNSCC. The TCGA-HNSCC cohort consisted of 31 normal individuals and 450 HNSCC patients, who were used for analyzing differential gene expression. Additionally, the clinical data of 456 HNSCC patients were downloaded, including information on survival time, survival status, age, gender, grade of pathology, clinical stage, and TNM stage. Furthermore, 442 somatic mutation samples were acquired for conducting tumor mutation burden (TMB) analyses.

### 2.2. Weighted Gene Co-Expression Network Analysis (WGCNA)

The “WGCNA” R package (R software version 4.2.0, the same below) was used to identify hub gene modules significantly associated with ubiquitination in HNSCC patients. Firstly, the “limma” R package was utilized to extract the expression profiles of ubiquitination-related genes from the TCGA-HNSCC cohort. Subsequently, a cluster analysis was performed on the extracted expression profiles, determining a soft threshold and clustering an adjacency matrix to identify genes within dynamic modules. Lastly, modules were clustered based on gene similarity and examined for correlation with clinical characteristics data, ultimately identifying a hub module. We selected the Pearson correlation coefficient to determine the strongest correlation between the modules and clinical sample data, which was chosen for further analysis.

### 2.3. Ubiquitination-Related Gene Signature Construction in HNSCC

First of all, genes in the hub module were iterated through one-to-one matching. We combined these gene pairs with TCGA-HNSCC transcriptome expression profiles and clinical characteristic data through the “limma” R package. Subsequently, we scored every gene pair by comparing their expression levels in the samples. In the same sample, a gene pair with a higher expression level of the former scored 1 point; otherwise, they scored 0 points. The advantage of constructing gene pairs lies in the utilization of an UbRG expression matrix consisting solely of 0 s and 1 s, allowing for a focus solely on the relative expression levels of genes without the need to consider the absolute amount of gene expression, the potential difference between batches, or to perform batch correction (only samples with a pair ratio of 0.2–0.8 were included; pair ratio = the total pair value/the total pair quantity) [26]. Subsequently, the HNSCC samples were randomly divided into two equal parts: the training set and testing set. The training set was utilized to identify the prognostic UbRG signature, while the testing set was employed for internal validation. Following this, significant pairs were recognized through univariate Cox regression analysis to establish a survival prediction signature. Ultimately, the risk score signature was developed using LASSO regression analysis, effectively reducing the risk of overfitting irrelevant variables. The model formula was constructed as follows:

$$\mathrm{Risk}\;\mathrm{score}=\sum_{i=1}^{N}\left(genepair’s\;score\times corresponding\;coefficient\right)$$

N stands for the number of genes. The LASSO Cox regression analysis was conducted by the “survival” and “glmnet” R packages.

### 2.4. Verification of the Prognostic UbRG Signature

The risk score for each individual in the TCGA-HNSCC cohort was computed, and, subsequently, HNSCC samples were divided into high-risk and low-risk groups based on the median risk score. To evaluate the predictive accuracy and robustness of the signature, time-dependent ROC analysis and Kaplan–Meier survival estimates were performed using the “timeROC” and “survival” R packages. The autonomy of the UbRG signature was assessed through univariate and multivariate Cox regression analyses using the “survival” and “glmnet” R packages. A *p*-value of less than 0.05 was considered statistically significant.

### 2.5. Development and Validation of the Nomogram

The nomogram, as a visual statistical tool, integrates various clinical and pathological factors to accurately predict the survival outcomes of cancer patients [27]. To gauge the 1-, 3-, and 5-year survival of HNSCC patients, variables including age, clinical stage, gender, TMN classification, pathological grade, and risk group were considered for constructing the nomogram. The “survival”, “regplot” and “rms” R packages were employed in this procedure. Subsequently, the prognostic significance of the nomogram was evaluated by calibration curves and ROC curves. The concordance index (C-index) represents the consistency between actual occurrence probability and prediction probability. A value of 1 indicates perfect prediction.

### 2.6. Gene Differential Expression Analysis of the Prognostic UbRG Signature

Differential expression analysis can identify genes with significant differences between groups while effectively mitigating experimental interference, errors, and other confounding factors [28]. The gene differential expression analysis was carried out to further validate the UbRGs of the prognosis model using the “limma” R package. Visualization of the analysis results was achieved through heatmap and boxplot generation using the “pheatmap”, “reshape2”, and “ggpubr” R packages. Statistical significance was determined by *p* < 0.05.

### 2.7. Gene Set Enrichment Analysis (GSEA)

The GSEA is a commonly used approach to assess the statistical significance and consistent variances among predefined gene sets in two different biological conditions [29]. To elucidate the potential pathways and biological processes associated with the UbRG signature in high-risk and low-risk groups, we performed a GSEA using a Kyoto Encyclopedia of Genes and Genomes (KEGG) pathway enrichment analysis and Gene Ontology (GO) functional annotation. This analysis was conducted utilizing the “clusterProfiler” and “enrichplot” R packages [30]. Initially, we applied the KEGG separately to both high-risk and low-risk groups. Then, the GO functional annotation facilitated identification of active genes within the predictive gene signature. Statistical significance was considered at *p* < 0.05 for filtering conditions.

### 2.8. Construction of Functional Interaction Networks of Model Genes, Transcription Factors, and Enhancer RNAs

The relationships between model genes and transcription factors (TFs), as well as between model genes and enhancer RNAs (eRNAs) (all DNA sequences associated with enhancer elements were included in the eRNA co-expression analysis, encompassing protein-coding genes that establish chromatin loops with promoters), were explored through co-expression analysis. This analysis aimed to identify TFs associated with model genes, model genes associated with TFs, model genes associated with eRNAs, and eRNAs associated with model genes. The TF data were obtained from the Cistrome platform (https://www.cistrome.org/, accessed on 20 January 2024), while the eRNA data were extracted from the published literature [31,32]. The procedure utilized R packages including “dplyr,” “ggpubr,” and “ggalluvial.” Significant thresholds for co-expression analysis were set at |cor| > 0.65, *p* < 0.001 for the relationship between model genes and TFs and |cor| > 0.5, *p* < 0.001 for the relationship between model genes and eRNAs.

### 2.9. Immune Landscapes Related to the Signature

To evaluate the distribution of 22 immune cell subpopulations within the tumor, we applied the CIBERSORT algorithm [33]. This technique estimates the relative distribution of cells in each sample based on the expression of cell-specific biomarkers, ensuring that the total of all calculated scores for immune cell types equals 1. Moreover, Spearman’s correlation analysis was performed to explore the association between gene expression levels and immune cell composition. The “survminer” R package was used to conduct a differential analysis of immunoinfiltrating cells. In addition, a single-sample gene set enrichment analysis (ssGSEA) was used to determine immune-related function scores for each sample [34]. Finally, we explored disparities in the expression levels of genes associated with immune checkpoints between the high-risk and low-risk groups.

### 2.10. Tumor Mutation Burden (TMB) Analysis

The TCGA-HNSCC cohort was utilized to perform a comparative analysis of the TMB, with the objective of evaluating the discrepancies in mutation profiles between high-risk and low-risk groups. Spearman’s correlation analysis was applied to examine the relationship between the TMB and risk score in each HNSCC sample. In order to investigate the correlation between TMB and prognosis, the TCGA-HNSCC cohort samples were categorized into high-TMB and low-TMB groups using a threshold of one mutation per megabase (MB) [35]. Survival analysis was conducted to assess the predictive performance of different TMB and risk score groups. Kaplan–Meier curves were generated using the “survival” and “survminer” packages in R.

### 2.11. Drug Sensitivity Prediction

To forecast the response of HNSCC patients to various chemotherapeutic agents, drug sensitivity analysis was conducted using the “oncoPredict” R package [36]. The “OncoPredict” R Package facilitates the integration of in vitro drug screening with in vivo drug and biomarker discovery by predicting drug responses in large cancer−molecular datasets. The GDSC2 dataset employed for this analysis was sourced from the Genomics of Drug Sensitivity in Cancer (GDSC) database (https://www.cancerrxgene.org/, accessed on 20 January 2024). The GDSC database offers open access to genomic data related to tumor therapy and is committed to identifying potential targets for tumor therapy in order to enhance cancer treatment. The GDSC database contains half maximal inhibitory concentration (IC50) values for nearly a thousand cell lines in response to various chemotherapy drugs. A total of 198 drugs were evaluated to compare the differences in drug sensitivity between high-risk and low-risk cohorts, utilizing the Wilcoxon test and considering a *p*-value of <0.001 as statistically significant.

### 2.12. Statistical Methods

**Figure 1:**
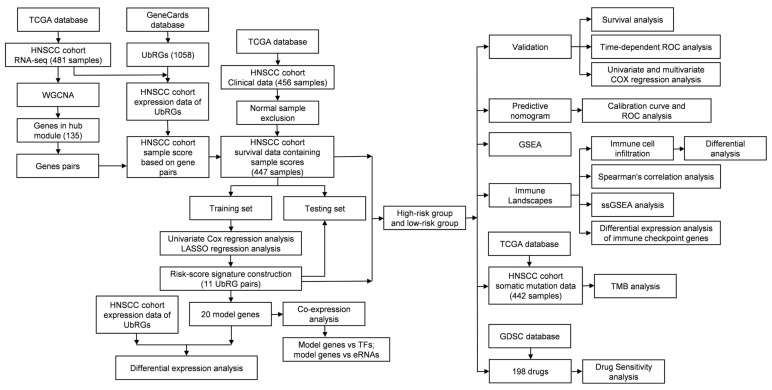
Block chart of the research methodology and data processing.

Statistical analyses were performed utilizing R software version 4.2.0 (The R Foundation for Statistical Computing; https://www.r-project.org/, accessed on 20 January 2024). All the script codes involved in our study have been compiled in Supplementary Materials-Script Codes. For gene expression analysis, the Mann–Whitney test was utilized, with statistical significance defined as a *p*-value below 0.05. The block chart illustrating research methods and data processing is depicted in Figure 1.

## 3. Results

### 3.1. Co-Expression Network of Ubiquitination-Related Genes

**Figure 2:**
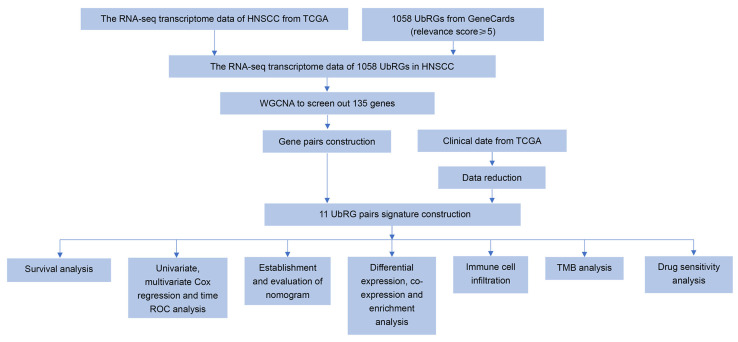
Flow diagram of the study. HNSCC, head and neck squamous cell carcinoma; TCGA, The Cancer Genome Atlas database; UbRGs, ubiquitination-related genes; WGCNA, Weighted Gene Co-expression Network Analysis; TMB, tumor mutation burden.

**Figure 3:**
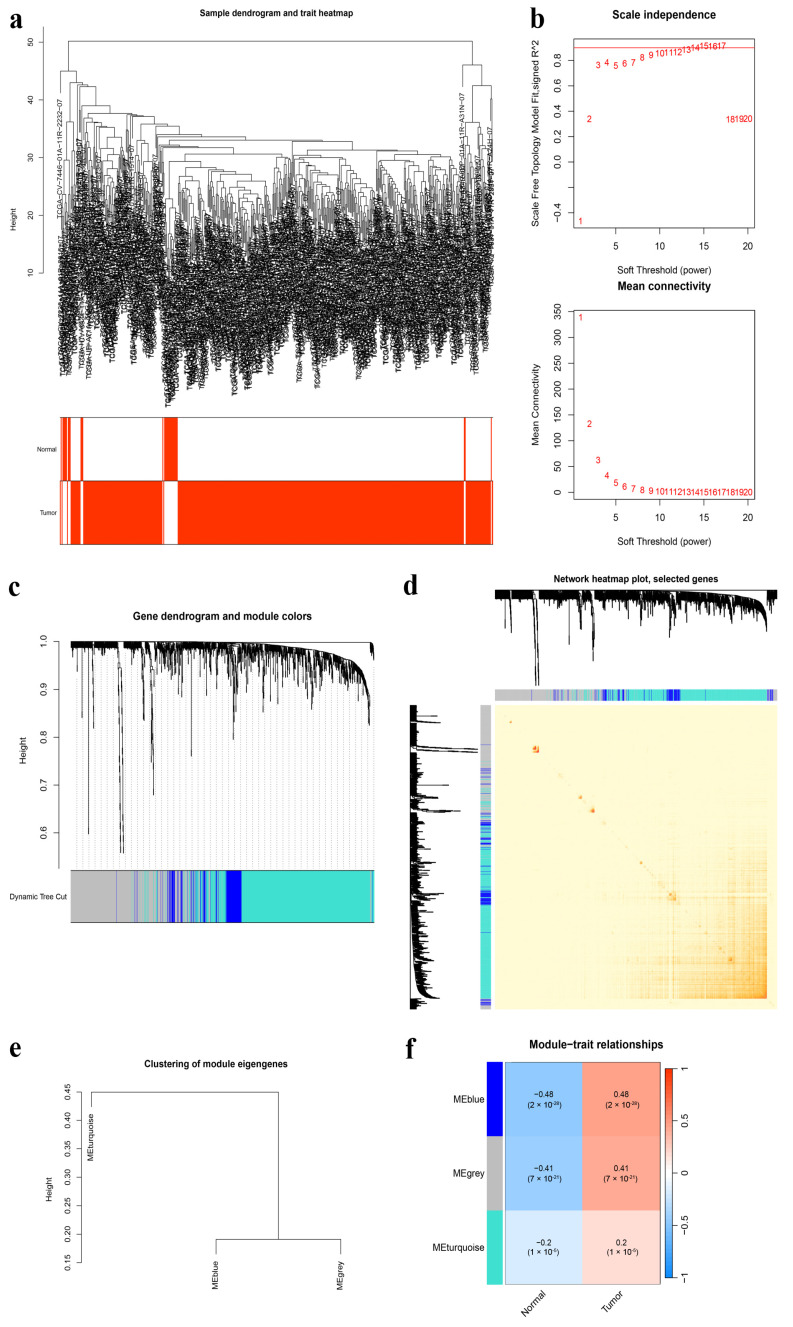
WGCNA. (**a**) The sample dendrogram and trait heatmap display the cluster analysis of UbRG expression data, with merged modules exhibiting similar expression profiles. (**b**) A scale-free network is constructed within the co-expression network, assessing scale independence and mean connectivity (β = 10). (**c**) A gene dendrogram and module colors are utilized to demonstrate UbRG clustering and module identification. Each gene is represented by a node. The vertical axis indicates the degree of topological difference between genes, with greater distances between vertical axes indicating stronger topological differences and weaker co-expression correlations. The horizontal axis represents different modules, each denoted by a distinct color. The Dynamic Tree Cut method is used to obtain modules through hierarchical clustering using average-linkages. (**d**) A network heatmap plot of the modules, based on 100 randomly selected genes. Red colors with increasing saturation indicate a higher level of overlap among functional modules. (**e**) The cluster dendrogram of module eigengenes. (**f**) Heat-map of the correlation between merged co-expression modules and clinic characteristics of HNSCC. The correlation scores are shown in each cell, and the significance values (*p*-value) are shown in the bracket below. WGCNA, Weighted Gene Co-expression Network Analysis; TCGA, The Cancer Genome Atlas database; HNSCC, head and neck squamous cell carcinoma; UbRGs, ubiquitination-related genes.

Figure 2 presents a flow chart that outlines the analytical procedures of this study. To identify and analyze gene modules exhibiting co-expression patterns, we employed a WGCNA, which also explored the association between the UbRG network and clinical features using expression profiles from the TCGA-HNSCC cohort. For clustering genes with significant topological overlap, we applied the topological overlap matrix (TOM) and refined the ensuing clustering dendrogram through the dynamic tree-cut method (Figure 3a). Our analysis determined a soft threshold power β = 10 by fulfilling two criteria: achieving a scale-free topology fit index of at least 0.90 and noting a marked reduction in connectivity measurements (Figure 3b). Subsequently, dynamic module identification based on the TOM matrix was performed, ensuring that each module contained no less than 50 genes. Three genetic modules, turquoise, blue and gray, were identified (Figure 3c). We generated a hierarchical clustering heat map to visualize the strong correlation among genes within these modules as well as their interconnection patterns (Figure 3d). The cluster dendrogram of module eigengenes provided further insights into the hierarchical relationships between these three co-expression modules (Figure 3e). Furthermore, by analyzing correlations between constructed modules and traits while considering their significance levels, we created a module−trait relationship heatmap. Our analysis revealed that the BLUE module exhibited a robust positive correlation with HNSCC (Cor = 0.48, *p* = 2 × 10^−28^) (Figure 3f), thus making it an ideal candidate for subsequent analyses as an HNSCC-correlated module.

### 3.2. Construction and Internal Validation of the UbRG Signature

**Figure 4:**
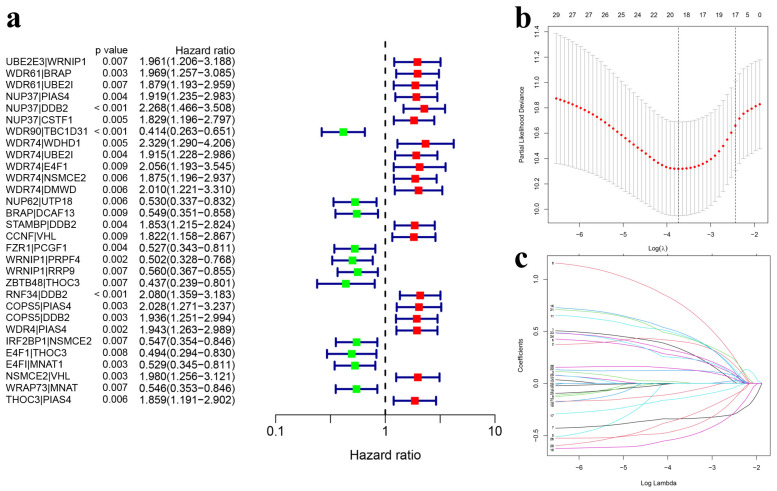
Development of the UbRG signature. (**a**) The forest plot illustrates 30 gene pairs linked to ubiquitination that show a significant association with overall survival, as identified by univariate Cox regression analysis. (**b**) The LASSO regression model reveals the partial likelihood deviance for the variables. Red dots on the graph indicate the values of partial likelihood deviance, while gray lines denote the standard error (SE); the left vertical dashed line marks the optimal value based on the minimum criteria, and the right vertical dashed line denotes the 1 − SE criteria. (**c**) LASSO coefficient profiles for the 30 ubiquitination-related gene pairs associated with prognosis in HNSCC. The horizontal axis represents the log-transformed value of the independent lambda and the vertical axis shows the coefficient of the independent variable. Each solid colored line represents a variable, namely a gene pair. UbRGs, ubiquitination-related genes; LASSO, least absolute shrinkage and selection operator; HNSCC, head and neck squamous cell carcinoma.

The hub module’s 135 genes were paired and then integrated with TCGA-HNSCC gene expression and clinical data. The survival analysis encompassed 447 patients, each with a follow-up period longer than one month. Using a univariate Cox regression analysis with a *p*-value threshold of <0.01, we identified 30 ubiquitination-related gene pairs with prognostic significance (Figure 4a). Subsequently, a LASSO Cox regression analysis with 10-fold cross-validation was utilized to determine the optimal penalty parameter, resulting in the establishment of the optimal prognostic signature. This analysis led to a final set of 18 nonzero coefficients when plotting a coefficient profile against the log l sequence (Figure 4b,c). Following this, multivariate Cox regression analysis identified 11 gene pairs (*UBE2E3*|*WRNIP1*, *WDR61*|*BRAP*, *NUP37*|*CSTF1*, *WDR90*|*TBC1D31*, *WDR74*|*WDHD1*, *WDR74*|*NSMCE2*, *CCNF*|*VHL*, *WRNIP1*|*PRPF4*, *RNF34*|*DDB2*, *WDR4*|*PIAS4*, and *E4F1*|*THOC3*) to construct our prognostic risk model. It should be noted that our developed prognostic UbRG pairs model is relatively independent, focusing solely on survival time and status without taking into account other clinical factors such as HPV status, gender, and TNM stage. The full names of these genes are listed in Table 1. Additionally, each patient’s risk score was calculated based on these eleven gene pairs using the formula specified in the methods section. Consequently, HNSCC samples were categorized into high-risk and low-risk groups based on their median risk score. The features of 447 TCGA clinical samples utilized for model construction are presented in Extended Data Table S1.

**Figure 5:**
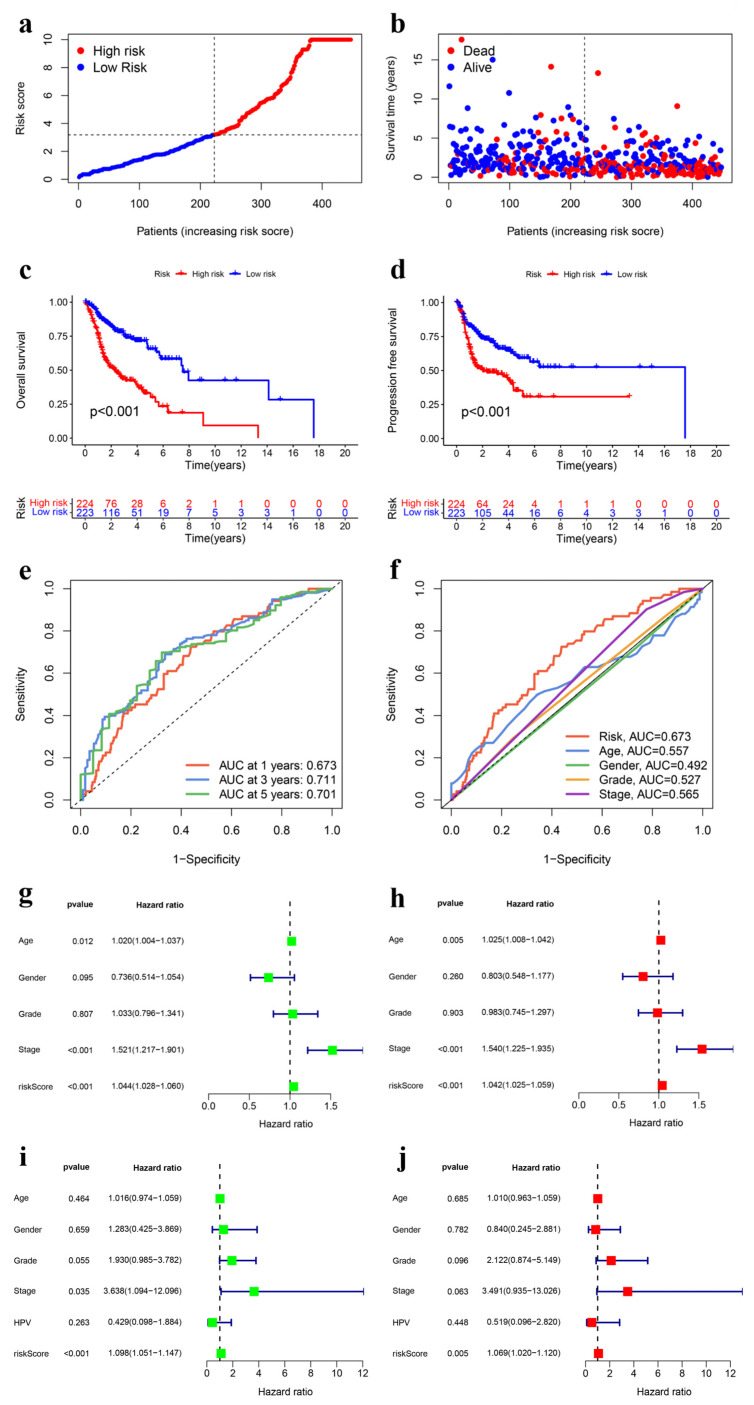
Verification of the prognostic UbRG signature. (**a**) demonstrates the distribution of risk scores in the TCGA-HNSCC cohort. (**b**) depicts the distribution of each patient’s survival status and associated risk score. (**c**) displays the overall survival Kaplan–Meier curves related to the prognostic UbRG signature. (**d**) shows the progression-free survival Kaplan–Meier curves associated with the prognostic UbRG signature. (**e**) illustrates time-dependent ROC analysis, indicating the predictive efficacy of the prognostic UbRG signature for overall survival. (**f**) compares 1-year ROC curves between the prognostic UbRG signature and clinical characteristics such as age, gender, pathological grade, and clinical stage. (**g**) Presents univariate Cox regression analysis that does not consider HPV status. (**h**) depicts multivariate Cox regression analysis without consideration of HPV status. (**i**) illustrates univariate Cox regression analysis that takes into account the HPV status. (**j**) demonstrates multivariate Cox regression analysis, taking into account the HPV status. UbRGs refer to ubiquitination-related genes, TCGA denotes The Cancer Genome Atlas database, HNSCC represents head and neck squamous cell carcinoma, and ROC stands for receiver operating characteristic. HPV stands for human papillomavirus.

Figure 5a,b visually depict the distribution of risk grades, survival status, and time patterns across the entire dataset. Various analyses were utilized to validate the prognostic significance of the risk score model. Kaplan–Meier survival analyses were employed to assess OS and progression-free survival (PFS) in HNSCC patients, revealing a notably worse prognosis for the high-risk group compared to the low-risk group (*p* < 0.001) (Figure 5c,d). Time-dependent ROC analysis was conducted to evaluate the effectiveness of the UbRG signature, yielding AUC values of 0.673, 0.711, and 0.701 for predicting 1-, 3-, and 5-year OS, respectively (Figure 5e). Importantly, our risk model exhibited superior predictive accuracy for one-year OS compared with other clinical prognostic variables such as age, gender, pathological grade, and clinical stage, as indicated by the highest AUC value. The AUC values for the risk score, age, gender, pathological grade, and clinical stage were 0.673, 0.557, 0.492, 0.527, and 0.565, respectively (Figure 5f). These results suggest that our risk score model is reliable for predicting outcomes in HNSCC patients. Furthermore, univariate/multivariate Cox regression analyses were performed to determine the substantial predictive value of our risk score model for HNSCC patients’ prognosis, resulting in significant hazard ratios (HRs) of 1.044 (95% CI: 1.028–1.060, *p* < 0.001) from the univariate Cox regression (Figure 5g) and an HR of 1.042 (95% CI: 1.025–1.059, *p* < 0.001) from the multivariate Cox regression (Figure 5h). These findings suggest that, in addition to our risk score model, age and clinical stage are also significant factors influencing outcomes in patients with HNSCC. The findings align with the prevailing clinical consensus. In conclusion, the developed UbRG-based risk score model demonstrates promising potential in accurately predicting outcomes for individuals diagnosed with HNSCC.

Human papillomavirus (HPV) infection is strongly associated with the development, progression, and prognosis of HNSCC. It is crucial to take HPV status into account when evaluating the prognosis of HNSCC patients. During the COX regression analysis, we compared our risk score model against various clinical characteristics, including age, gender, pathological grade, clinical stage, and HPV status. To maintain the accuracy of the COX regression analysis, we excluded any samples with incomplete clinical data. Out of the initial 456 samples in the clinical dataset, only 78 were used for the COX regression analysis due to these criteria. The analysis indicated that only our risk score model was a significant predictor of HNSCC patient outcomes (*p* < 0.05), while other clinical features were not (Figure 5i,j). We believe that this result may lack reliability due to the limited sample size. Consequently, the HPV status was not included in the subsequent development of the nomogram.

### 3.3. Development and Validation of a Prognostic Nomogram

**Figure 6:**
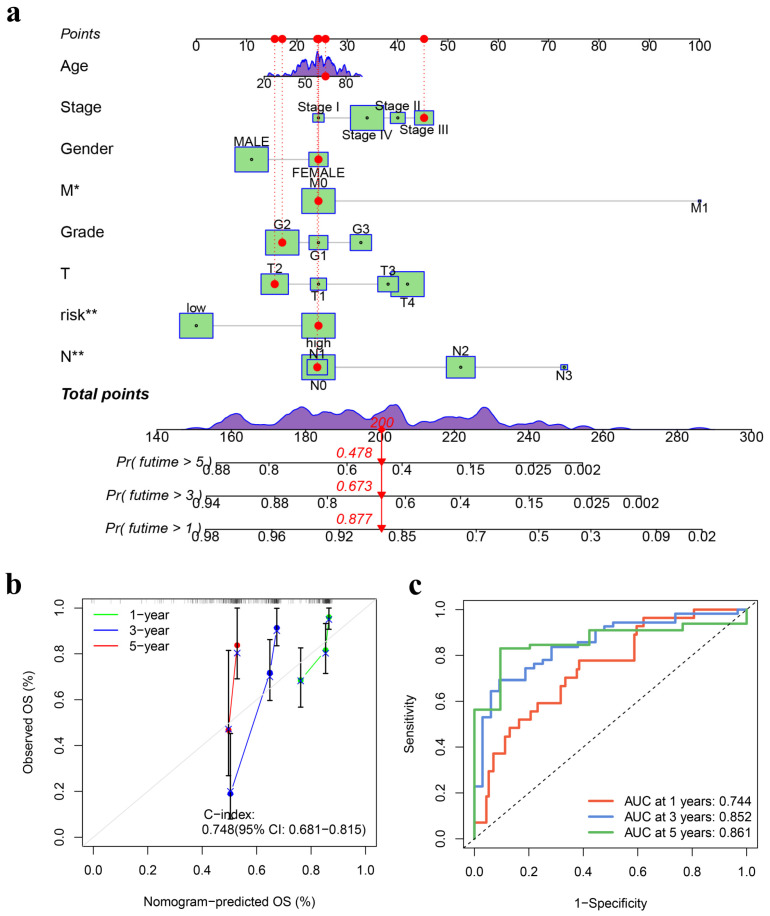
Development and verification of a nomogram. (**a**) A nomogram was constructed to predict the overall survival of HNSCC patients at 1, 3, and 5 years. The clinical characteristics of each patient were located on the variable row and connected with the points row through a vertical line, assigning a corresponding value of points to each variable. This process was repeated for all variables, and the total points for the seven prognostic factors were calculated. A vertical line was then drawn downward from the total points row to determine the predicted overall survival at 1, 3, and 5 years for HNSCC patients. * and ** denote *p* < 0.05 and *p* < 0.01, respectively. (**b**) The calibration plot was used for the internal validation of the nomogram’s overall survival prediction. The “ideal” line, represented by the gray line, indicates a perfect match between the observed and nomogram-predicted survival rates. The dots represent the apparent predictive accuracy, while the blue X represents the estimated values after bootstrap correction. (**c**) The ROC curves demonstrate the high predictive efficiency of the nomogram. HNSCC, head and neck squamous cell carcinoma; ROC, receiver operating characteristic.

A personalized nomogram was developed in the study for patients diagnosed with HNSCC, integrating eight distinct prognostic variables (age, gender, pathological grade, clinical stage, TNM, and risk score) to predict individualized OS rates accurately at 1-, 3-, and 5-year time frames (Figure 6a). Validation of the nomogram’s predictive accuracy was performed using calibration plots, revealing a close correspondence with actual observed survival rates at these specific intervals (Concordance index: 0.748, 95% CI: 0.681–0.815) (Figure 6b). Assessment of the nomogram’s predictive performance in terms of survival outcomes involved the use of estimated probability ROC curves to evaluate its sensitivity and specificity. Noteworthy results included remarkable AUC values of 0.744 for one-year survival rate prediction, 0.852 for three-year survival rate prediction, and an exceptional value of 0.861 for five-year survival rate prediction (Figure 6c).

### 3.4. External Analysis of the UbRG Signature

**Figure 7:**
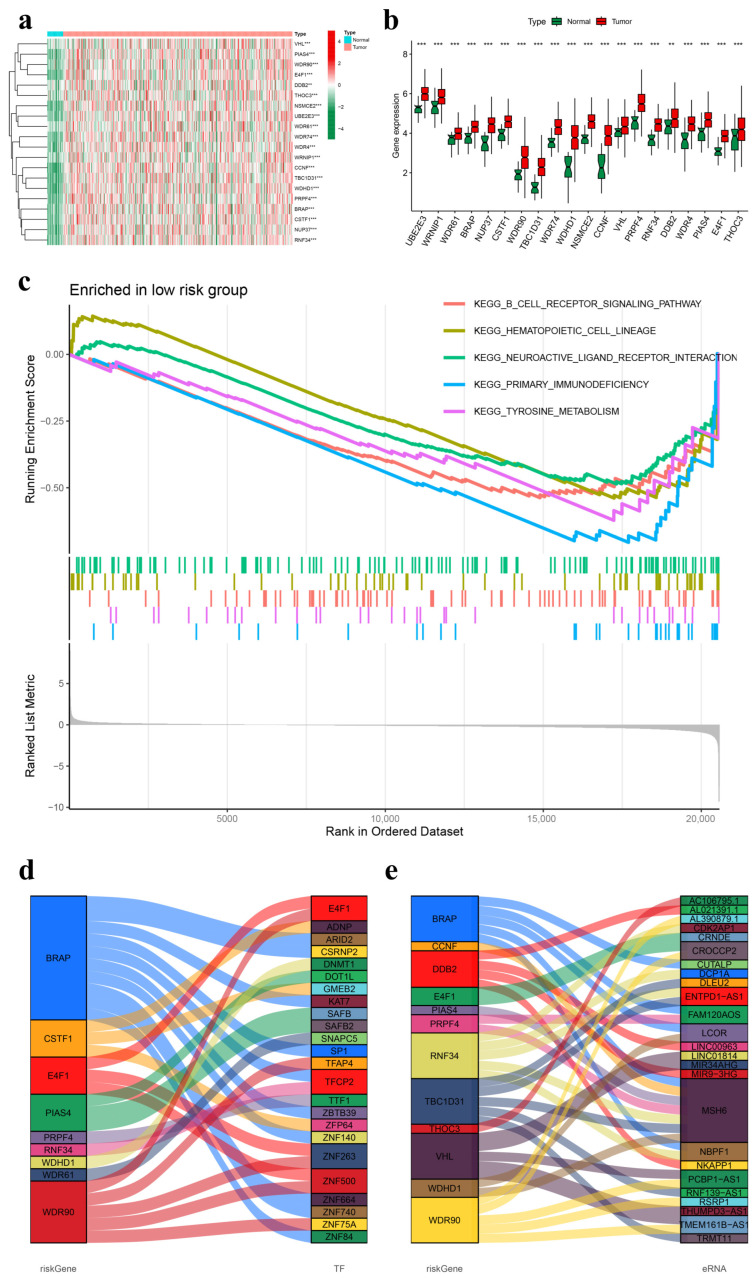
External evaluation of the UbRG signature. (**a**) A heatmap depicting 20 differentially expressed genes within the prognostic UbRG signature is shown. (**b**) Boxplot visualizations of these 20 differentially expressed genes within the prognostic UbRG signature are provided. Asterisks ** and *** denote the significance levels of 0.01 and 0.001, respectively. (**c**) Enrichment plots highlight gene enrichment in the low-risk group for hematopoietic cell lineage (orange), neuroactive ligand receptor interaction (green), tyrosine metabolism (pink), B cell receptor signaling pathway (red), and primary immunodeficiency (blue). No gene enrichment was detected in the high-risk group. (**d**) A Sankey diagram depicts the relationship between nine risk genes in the prognostic UbRGs (on the **left**) and 24 transcription factors (TFs) (on the **right**). The height of the boxes represents the proportion of risk genes and TF types, with lines indicating their associations. (**e**) Another Sankey diagram illustrates the connections between 12 risk genes in the prognostic UbRGs (**left**) and 25 enhancer RNAs (eRNAs) (**right**). UbRGs stands for ubiquitination-related genes; TF refers to transcription factor; eRNA signifies enhancer RNA.

Differential expression analysis was conducted to compare the gene expression levels between tumor samples and normal samples, focusing on the genes comprising the UbRG signature. A comprehensive assessment of 20 genes revealed a consistent and significant upregulation in all 20 genes across the tumor samples (Figure 7a,b).

The GSEA findings related to the UbRG signature indicated that the low-risk group displayed enrichment in various pathways, such as the B cell receptor signaling pathway (red), hematopoietic cell lineage (orange), neuroactive ligand receptor interaction (green), primary immunodeficiency (blue), and tyrosine metabolism (pink) (Figure 7c). Conversely, no pathway was enriched in the high-risk group.

The construction of functional interaction networks involving model genes was performed by integrating transcription factors (TFs) and enhancer RNAs (eRNAs). A total of 1639 TFs were obtained from the Cistrome platform. Co-expression analysis revealed significant correlations between nine types of model genes and 24 types of TFs, such as E4F1, TFCP2, ZNF263, and ZNF500 (Figure 7d). Additionally, a total of 1584 eRNAs were extracted for co-expression analysis with model genes, revealing strong associations between 12 types of model genes and 25 types of eRNAs, including MSH6, NBPF1, LCOR, and TMEM161B-AS1 (Figure 7e).

### 3.5. Immune Landscapes Related to the Signature

**Figure 8:**
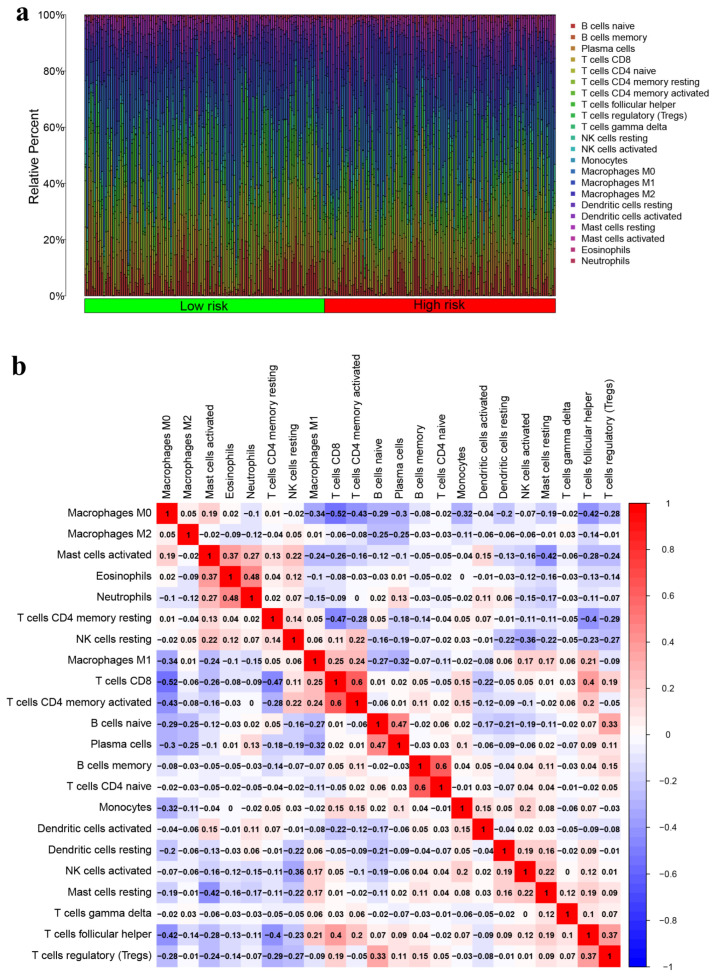
Analysis of immune cell infiltration. (**a**) The distribution of 22 immune cell subsets in HNSCC is presented, with the percentage of each type of immune cell being shown on the vertical axis and the TCGA-HNSCC samples being displayed on the horizontal axis. (**b**) Co-expression patterns among immune cells are depicted using a color scheme, where red signifies a positive correlation and blue signifies a negative correlation. TCGA refers to The Cancer Genome Atlas database, while HNSCC stands for head and neck squamous cell carcinoma.

**Figure 9:**
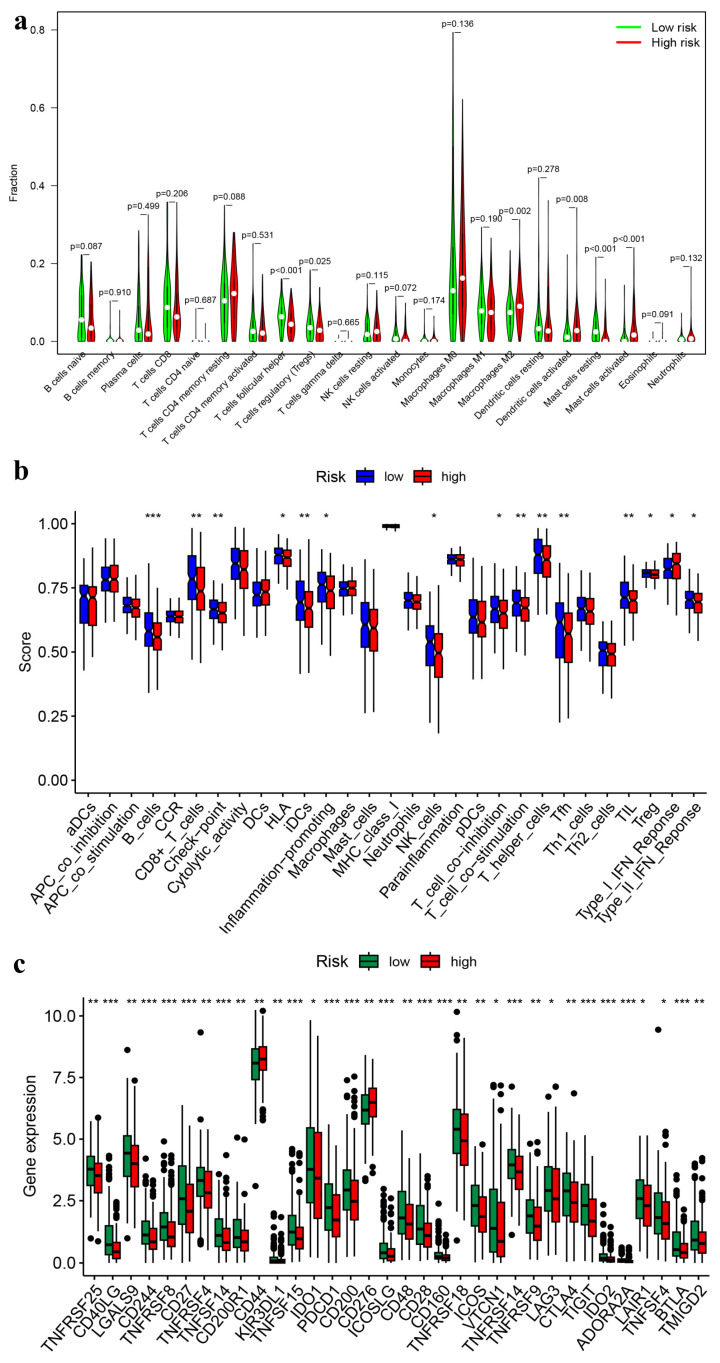
Immune landscapes associated with the prognostic signature of UbRGs. (**a**) The violin plot depicts variations in immune infiltration between the high-risk and low-risk groups in HNSCC. The statistical significance values (*p*-value) are indicated above. (**b**) Variations in immune-related function between the high-risk and low-risk groups in HNSCC are presented in the box plot. (**c**) The box plot demonstrates differences in the expression levels of genes related to immune checkpoints between the high-risk and low-risk groups in HNSCC. The significance levels are denoted by *, **, and *** correspond to 0.05, 0.01, and 0.001, respectively. Here, UbRGs refer to ubiquitination-related genes and HNSCC stands for head and neck squamous cell carcinoma.

The CIBERSORT algorithm was utilized to examine the differences in immune cell composition between high-risk and low-risk HNSCC groups, focusing on 22 specific immune cell types (Figure 8a). The co-expression patterns of these immune cells were visually depicted, showing weak to moderate correlations in their proportions within HNSCC tissues (Figure 8b). Through heatmap analysis, it was found that activated memory CD4+ T cells and CD8+ T cells had the highest positive correlation. Similarly, naïve CD4+ T cells and memory B cells showed a strong positive correlation. On the other hand, the most substantial negative correlation was between CD8+ T cells and M0 macrophages. Additionally, a violin plot indicated that M2 macrophages (*p* = 0.002), activated dendritic cells (*p* = 0.008), and activated mast cells (*p* < 0.001) were more prevalent in the high-risk group. In contrast, follicular helper T cells (*p* < 0.001), regulatory T cells (Tregs) (*p* = 0.025), and resting mast cells (*p* < 0.001) exhibited lower infiltration levels in the high-risk group compared to the low-risk group (Figure 9a). Research has demonstrated that selective activation of tumor-associated macrophages (M2) can support tumor cell growth, while the metabolic changes in anti-tumor macrophages resemble those of classically activated (M1) macrophages [37,38]. The presence of elevated levels of M2 macrophages in our high-risk group is in line with this finding.

Next, we conducted an ssGSEA to comprehensively evaluate immune-related functions to discern differences between the high- and low-risk groups more effectively. Our findings revealed upregulation of crucial immune-related pathways such as T cell co-inhibition (*p* < 0.05), T cell co-stimulation (*p* < 0.01), and Type II interferon (IFN) response (*p* < 0.05) in the low-risk group; conversely, the Type I IFN response (*p* < 0.05) was found to be upregulated in the high-risk group (Figure 9b). Upregulation of immune response pathways can heighten the aggressiveness of the host immune system against tumor cells, enhancing their killing rates and inhibition while limiting tumor growth.

Finally, a differential analysis of genes related to immune checkpoints was performed. Immune checkpoint refers to programmed death receptors and their ligands, including stimulating and inhibiting immune receptors [39]. Expression of immune checkpoints can impact the host immune system and form the basis for tumor immunotherapy. Activation of stimulatory immune receptors can impede tumor growth by stimulating immune cells, whereas inhibitory immune receptors trigger signaling pathways that inhibit the immune cell response to tumor cells and hinder tumor eradication. The result of differential analysis unveiled that the expression levels of thirty-two immune checkpoint-related genes [including tumor necrosis factor receptor superfamily member 25 (*TNFRSF25*), *CD40LG*, and *LGALS9*] were significantly upregulated in the low-risk group. Stimulating immune receptors’ TNFRSF25 can enhance the eradication of tumor cells by T cells [40], and CD40LG activates immune cells and enhances inflammatory responses [39]. On the other hand, the analysis findings also indicated that CD44 and CD276 were upregulated in the high-risk group (Figure 9c). Like programmed cell death ligand 1 (PD-L1), inhibiting immune receptors CD276 can inhibit T cell proliferation, thereby dampening their ability to kill off tumors [41]. CD44 is involved in heterogeneous adhesion, which promotes vascular invasion and distant metastasis [42]. Taken together, the results suggest that these UbRGs may exert their prognostic influence by manipulating the activity of immune cells in HNSCC.

### 3.6. TMB Analysis

**Figure 10:**
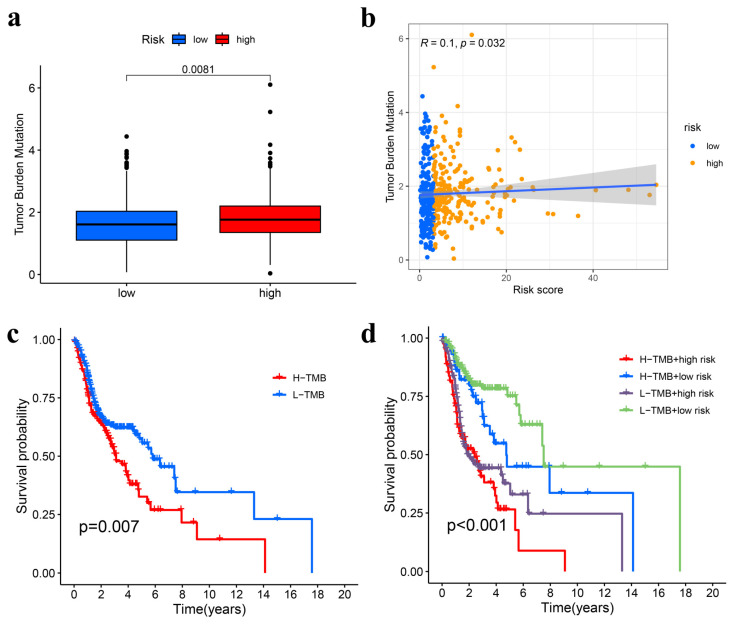
Analysis of TMB. (**a**) The box plot illustrates that TMB is elevated in the high-risk group of HNSCC patients, with significance values (*p*-values) indicated numerically. (**b**) A correlation analysis between the risk score and TMB within the TCGA-HNSCC cohort is presented. (**c**) Kaplan–Meier survival curves indicate that the low-TMB group exhibits a higher overall survival probability. (**d**) Combined Kaplan–Meier survival curves for TMB and risk score show that patients categorized as low-TMB and low-risk have the best overall survival probability. TMB, tumor mutation burden; TCGA, The Cancer Genome Atlas database; HNSCC, head and neck squamous cell carcinoma; H-TMB, high tumor mutation burden; L-TMB, low tumor mutation burden.

The TMB data for 439 HNSCC samples were obtained from TCGA using VarScan2. The high-risk group exhibited a significantly elevated TMB compared to the low-risk group (*p* = 0.0081) (Figure 10a). A very weak positive linear correlation between the risk score and TMB was identified through correlation analysis (R = 0.1, *p* = 0.032) (Figure 10b). Based on a threshold of one mutation per megabase, patients were divided into high-TMB (*n* = 378) and low-TMB (*n* = 61) categories. Survival analysis indicated that patients with low-TMB had longer overall survival (OS) than those with high-TMB (*p* = 0.007) (Figure 10c). Moreover, the OS was significantly better for patients in the low-TMB and low-risk group compared to those in both the high-TMB and high-risk groups (*p* < 0.001) (Figure 10d).

### 3.7. Drug Responses in High- and Low-Risk Groups of HNSCC

**Figure 11:**
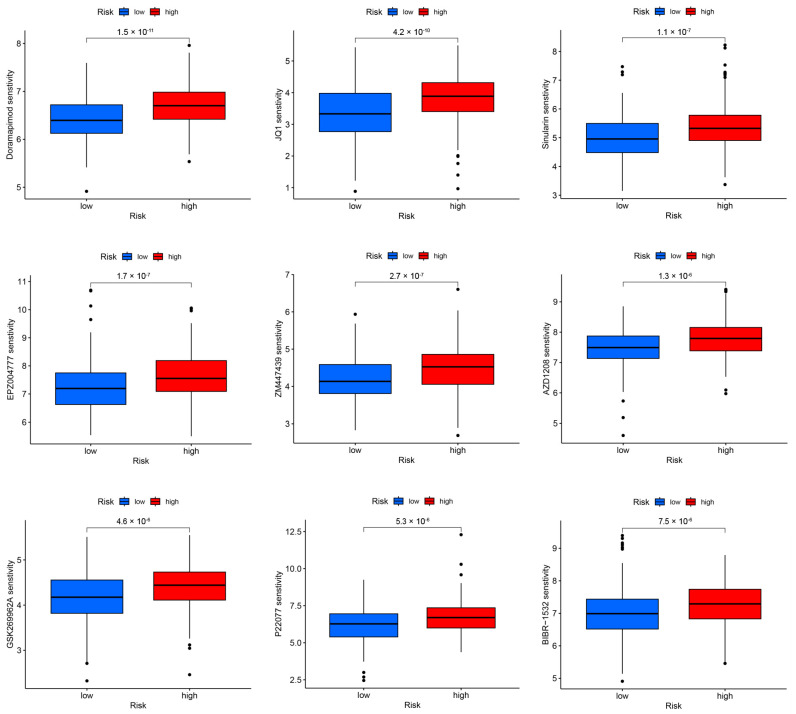
Boxplot shows a better chemotherapeutic sensitivity of nine drugs in the low-risk group in HNSCC. The number represents the significance value (*p*-value). HNSCC, head and neck squamous cell carcinoma.

The drug sensitivity analysis revealed that there were 42 drugs exhibiting statistically significant differences in response between the high- and low-risk groups (*p* < 0.001) (Extended Data Table S2). The lower the sensitivity score, the higher the level of sensitivity. Several chemotherapeutic agents, including doramapimod, JQ1, sinularin, EPZ004777, ZM447439, AZD1208, GSK269962A, P22077, and BIBR-1532 demonstrated lower sensitivity scores in the low-risk group (Figure 11), suggesting a potential sensitivity of these drugs towards HNSCC patients belonging to this group.

## 4. Discussion

HNSCC arises in the upper respiratory and digestive tracts, including the oral cavity, oropharynx, nasopharynx, hypopharynx, larynx, and paranasal sinuses, which makes it a heterogeneous group of carcinomas composing several distinct biological and clinical entities [43,44]. The inherent complexity of the genome, transcriptional processes, and molecular mechanisms significantly influences the response to treatment and prognosis of HNSCC [45]. With the emergence of precision medicine [46], the concept of individualized treatment has become particularly prominent in cancer, necessitating reliable prognostic tools to facilitate accurate prediction of outcomes in HNSCC. The accessibility of next generation sequencing data through public databases, such as TCGA and the Gene Expression Omnibus (GEO), holds immense potential for biomarker discovery and prognostic prediction in malignant tumors. Recently, mounting evidence suggests that UbRGs play a pivotal role in the pathogenesis of HNSCC. For instance, in patients with HNSCC, the overexpression of ubiquitin-specific protease 7 (*USP7*) is correlated with the upregulation of transcriptional co-activators with PDZ-binding motif (TAZ), increased tumor aggressiveness, and unfavorable prognosis [47]; BRCA2 and CDKN1A interacting protein β (BCCIPβ) interacts with E6, facilitating p53 ubiquitination in HPV-positive HNSCC and exerting a carcinogenic role in HPV16-positive HNSCC [48]; and the deubiquitylating enzyme ubiquitin-specific protease 9X (USP9X) facilitates the proliferation of head and neck cancer cells via activation of the mechanistic target of the rapamycin (mTOR) pathway [49]. Furthermore, the prognostic significance of UbRGs has been demonstrated in various cancers, including pancreatic duct adenocarcinoma [50], pancreatic cancer [51], colon cancer [52], and lung adenocarcinoma [53]. However, no studies have thus far reported on the prognostic potential and therapeutic significance of UbRG signatures for HNSCC.

In this study, we employed bioinformatics analysis to screen 11 ubiquitination-related gene pairs as a novel prognostic feature. To our knowledge, these UbRG signatures are the first to be established in HNSCC. Some UbRGs that build gene pairs have been researched; for example, the expression of endogenous hypoxic markers, which serve as prognostic indicators for HNSCC, is significantly inhibited by DDB2 [54]. The expression of DDB2 in tumors can serve as a direct predictor for the progression of interstitial HNSCC [55]. The upregulation of WDR4 has been reported in HNSCC and is associated with a poor prognosis [56]. The ubiquitin-conjugating enzyme UBE2E3 is highly conserved across metazoans. Depletion of UBE2E3 in cells leads to senescence even though it is independent of significant DNA damage [57]. The ubiquitin ligase BRAP exerts regulatory control over hepatocyte morphology and turnover within the Hippo pathway [58]. Given the current inadequate studies on UbRGs in HNSCC, it is imperative to further explore the molecular functionality and underlying mechanisms of UbRGs.

According to our risk score model, low-risk patients exhibit longer survival time and a more favorable prognosis. The AUC value of 0.673 surpasses that of other predictors, including age (0.557), gender (0.492), grade (0.527), and stage (0.565). This indicates that the predictive performance of this signature is more reliable and stable. In addition to the risk score model, both stage and age were identified as significant predictors, suggesting that the developed features can serve as independent prognostic factors for predicting the outcome of HNSCC patients. We have also developed a nomogram for the prediction of OS in patients with HNSCC. Both the risk model and nomogram exhibit excellent accuracy, reliability, and sensitivity, as demonstrated by the ROC curve analysis and calibration curve. Furthermore, functional analysis has revealed a significant association between differentially expressed UbRGs and HNSCC, highlighting the crucial role of ubiquitination dysregulation in cancer initiation and progression.

One of the main components found in tumor lesions is immune cells, which infiltrate different tumor microenvironments with varying types and functions. The response of the tumor microenvironment to specific therapies, especially cancer immunotherapy, is often determined by the composition of immune cells [59]. Therefore, analyzing the relationship between immune cell infiltration and HNSCC gene characteristics can provide insights into the status of the HNSCC immune microenvironment. In this study, we utilized the CIBERSORT algorithm to evaluate the relative abundance of 22 distinct immune cell types in HNSCC samples. Our results indicate that the low-risk group displayed a higher quantity of immune cells, increased immune function scores, and enhanced activation of various immune checkpoints, including cytotoxic T-lymphocyte associated protein 4 (CTLA4), CD28, and lymphocyte activating 3 (LAG3). These findings suggest a positive correlation between immune cell activation and a favorable prognosis.

Our findings demonstrate a significant correlation between high TMB and unfavorable prognosis in HNSCC patients, consistent with previous investigations [60,61]. Notably, TMB exhibits promising prognostic value as a standalone marker or when integrated into the risk score model for HNSCC patients. TMB is defined as the total number of non-synonymous somatic mutations per megabase (Mb) of the tumor genome and serves as an indicator of both tumor heterogeneity and immunogenicity [62]. The utility of TMB as a biomarker has been well established in predicting the clinical response to immune checkpoint inhibitors in various solid tumors, such as gastrointestinal cancer [63], breast cancer, non-small cell lung cancer, rectal cancer, and melanoma [64,65]. Currently, TMB has been reported to have a significant association with the response to pembrolizumab in patients with recurrent/metastatic HNSCC [66].

Immunotherapy has emerged as a promising therapeutic approach for managing HNSCC. However, the efficacy of these expensive interventions remains limited to a subset of patients [67]. In addition to cytotoxic chemotherapeutic agents, epidermal growth factor receptor and tyrosine kinase inhibitors, two immune checkpoint inhibitors targeting programmed cell death protein 1 (pembrolizumab and nivolumab), are currently used for patients with recurrent or metastatic HNSCC, but their clinical efficacy is still limited [67]. Based on the results of drug sensitivity analysis, we have identified several potential personalized precision drugs, such as doramapimod, JQ1, and sinularin, to improve treatment outcomes. However, further investigation is required to validate the efficacy and potential for individualization of these drugs.

There are several limitations that should be taken into account in this study. Firstly, given the heterogeneity of HNSCC, a separate and comprehensive analysis is warranted to consider its subtypes. Secondly, although our study heavily relies on data obtained from TCGA, it is essential to validate the results using other datasets for further confirmation. Thirdly, additional in vitro and in vivo experiments are necessary to corroborate our findings. Lastly, it is worth noting that genetic testing of the genes included in our model may incur substantial costs.

## 5. Conclusions

There is a lack of risk prediction models adopting ubiquitination-related genes in HNSCC. This research is a starting point. In summary, we developed a robust model that aggregates 11 ubiquitination-related gene pairs for risk stratification and established a nomogram that accurately predicts overall survival in patients with head and neck squamous cell carcinoma. The close correlation between risk score, immune infiltration, and the progression of HNSCC were unequivocally validated. These findings provided compelling molecular and bioinformatic evidence, shedding new light on the pivotal role of immune cells in tumorigenesis and highlighting the potential for precision personalized therapy in HNSCC. In the future, when gene sequencing technology is widely used in clinical cancer patients, our risk score model can aid clinicians in accurately evaluating patient prognosis. The tumor-immune microenvironment is a key focus of tumor research, and the screening of tumor targeted drugs has always been a priority. Since our study results are based on big data mining technology in the database, relevant in vitro experiments and animal experiments can be conducted to validate the findings of this study, and further exploration of mechanisms can be carried out.

## Figures and Tables

**Table 1:** Abbreviations of genes comprising prognostic signature.

Abbreviations	Full Names
*UBE2E3*	Ubiquitin-conjugating enzyme E2 E3
*WRNIP1*	Werner helicase-interacting protein 1
*WDR61*	WD repeat domain 61
*BRAP*	BRCA1-associated protein
*NUP37*	Nucleoporin 37
*CSTF1*	Cleavage stimulation factor 1
*WDR90*	WD repeat domain 90
*TBC1D31*	TBC1 domain family member 31
*WDR74*	WD repeat domain 74
*WDHD1*	WD repeat and high-mobility group box DNA binding protein 1
*NSMCE2*	NSE2 (MMS21) homolog, SMC5-SMC6 complex SUMO ligase
*CCNF*	Cyclin F
*VHL*	Von Hippel−Lindau tumor suppressor
*PRPF4*	Pre-mRNA splicing tri-snRNP complex factor PRPF4
*RNF34*	Ring finger protein 34
*DDB2*	Damage specific DNA binding protein 2
*WDR4*	WD repeat domain 4
*PIAS4*	Protein inhibitor of activated STAT 4
*E4F1*	E4F transcription factor 1
*THOC3*	THO complex subunit 3

## Data Availability

The original data presented in the study are openly available in the TCGA repository (https://portal.gdc.cancer.gov/, accessed on 15 January 2024), the GeneCards database (https://www.genecards.org/, accessed on 15 January 2024), the Cistrome platform (https://www.cistrome.org/, accessed on 20 January 2024) and the GDSC database (https://www.cancerrxgene.org/, accessed on 20 January 2024).

## Supplementary Materials

The following supporting information can be downloaded at: https://www.mdpi.com/article/10.3390/genes15070880/s1, Table S1. Characteristics of TCGA clinical samples in the UbRGs signature construction; Table S2. A list of 42 potential therapeutic agents has been predicted based on drug sensitivity analysis. R script code.
