# Supplementary material for: Ubiquitination-Related Gene Signature, Nomogram and Immune Features for Prognostic Prediction in Patients with Head and Neck Squamous Cell Carcinoma
Source: Genes (Basel). 2024 Jul 4;15(7):880. doi: 10.3390/genes15070880 (PMC11276148; doi:10.3390/genes15070880)
Supplement: Supplementary file 1 [file genes-15-00880-s001.zip › genes-2992241-supplementary.pdf]

Extended Data Table S1. Characteristics of TCGA clinical samples in the UbRGs signature construction.

ID	Survival time (day)	Survival state	Age	Gender	Patholo-gical grade	Clinical stage	T	N	M	HPV status	Risk-score	Risk group	Set group
TCGA-F7-8298	995	0	58	Male	G1	Stage I	T1	N0	M0	-	14.74723	High	Testing
TCGA-CV-A45Z	1466	1	75	Male	G3	-	T1	N0	M0	-	7.926787	High	Testing
TCGA-BA-A4II	918	0	46	Male	G1	Stage II	T2	N0	M0	-	7.212905	High	Testing
TCGA-F7-A61V	759	0	54	Male	G1	Stage II	T2	N0	M0	Negative	3.137531	High	Testing
TCGA-D6-8568	759	0	62	Male	G2	Stage II	T2	N0	M0	-	12.06513	High	Testing
TCGA-CQ-A4C6	1353	0	63	Male	G2	Stage II	T2	N0	M0	-	8.185341	High	Testing
TCGA-CV-A6JZ	714	0	68	Male	G2	Stage II	T2	N0	M0	-	3.137531	High	Testing
TCGA-IQ-7630	485	0	49	Male	G3	Stage II	T2	N0	M0	-	5.711611	High	Testing
TCGA-F7-A50J	947	0	67	Female	G2	Stage III	T3	N0	M0	-	14.43005	High	Testing
TCGA-CR-7364	1435	0	66	Male	G2	Stage III	T3	N0	M0	-	4.672831	High	Testing
TCGA-UF-A71B	1506	0	50	Male	G2	Stage IVA	T4	N0	M0	-	4.039041	High	Testing
TCGA-CV-A463	23	1	82	Female	G2	Stage IVA	T4a	N0	M0	-	21.49116	High	Testing
TCGA-CN-4738	436	1	53	Male	G2	Stage IVA	T4a	N0	M0	-	4.241203	High	Testing
TCGA-UF-A718	1971	0	62	Male	G2	Stage IVA	T4a	N0	M0	-	4.170785	High	Testing
TCGA-QK-A6VB	641	0	66	Male	G2	Stage IVA	T4a	N0	M0	-	5.00101	High	Testing
TCGA-CR-7390	1508	0	67	Male	G2	Stage IVA	T4a	N0	M0	Negative	8.506496	High	Testing
TCGA-F7-A50I	92	0	72	Male	G2	Stage IVA	T4a	N0	M0	-	5.338649	High	Testing
TCGA-IQ-A61H	1138	0	76	Male	G2	Stage II	T2	N0	MX	-	4.525229	High	Testing
TCGA-CN-A498	773	1	61	Female	G1	Stage III	T3	N0	MX	-	5.547619	High	Testing
TCGA-KU-A66T	552	0	53	Female	G2	Stage IVA	T4	N0	MX	-	14.65332	High	Testing
TCGA-CN-A49A	526	1	60	Male	G2	Stage IVB	T4b	N0	MX	-	3.937022	High	Testing
TCGA-CV-7238	2727	0	69	Female	G2	Stage II	T2	N0	-	-	4.539068	High	Testing

TCGA-D6-6823	701	0	50	Male	G2	Stage II	T2	N0	-	-	6.739576	High	Testing
TCGA-WA-A7GZ	625	1	58	Male	G2	-	T2	N0	-	-	4.90549	High	Testing
TCGA-CV-6941	342	1	51	Male	G2	Stage III	T3	N0	-	-	6.739576	High	Testing
TCGA-CV-7247	577	1	55	Male	G2	Stage III	T3	N0	-	-	5.678326	High	Testing
TCGA-CV-7250	2900	1	64	Male	G1	Stage IVA	T4a	N0	-	-	5.711611	High	Testing
TCGA-BA-5151	722	0	72	Male	G1	Stage IVA	T4a	N0	-	-	8.365259	High	Testing
TCGA-CV-7255	64	1	32	Female	G2	Stage IVA	T4a	N0	-	-	4.08108	High	Testing
TCGA-CN-4727	1560	0	56	Male	G2	Stage IVA	T4a	N0	-	Negative	11.80563	High	Testing
TCGA-CV-7430	495	1	56	Male	G2	Stage IVA	T4a	N0	-	-	7.215955	High	Testing
TCGA-CV-7245	797	0	62	Male	G2	Stage IVA	T4a	N0	-	-	3.812662	High	Testing
TCGA-CN-5355	1278	0	64	Male	G2	Stage IVA	T4a	N0	-	-	3.241631	High	Testing
TCGA-CN-4723	1699	0	67	Male	G2	Stage IVA	T4a	N0	-	-	3.201717	High	Testing
TCGA-CV-7421	2	1	76	Male	G2	Stage IVA	T4a	N0	-	-	21.13434	High	Testing
TCGA-HD-7229	1027	0	60	Male	G3	Stage IVA	T4a	N0	-	-	5.130403	High	Testing
TCGA-D6-A6EM	232	0	65	Female	G2	Stage III	T2	N1	M0	-	4.300767	High	Testing
TCGA-CQ-7071	1311	0	76	Female	G2	Stage III	T2	N1	M0	-	12.26883	High	Testing
TCGA-CQ-A4CI	950	0	73	Male	G3	Stage III	T2	N1	M0	-	6.54607	High	Testing
TCGA-F7-A620	543	0	47	Male	G1	Stage III	T3	N1	M0	-	7.926787	High	Testing
TCGA-UF-A7JA	2265	0	66	Female	G2	Stage IVA	T4a	N1	M0	-	4.90549	High	Testing
TCGA-BB-A5HZ	827	0	65	Male	G2	Stage IVA	T4a	N1	M0	-	12.06513	High	Testing
TCGA-QK-A8Z8	171	1	60	Female	G2	Stage IVC	T3	N1	M1	-	4.525229	High	Testing
TCGA-HD-8635	695	0	61	Female	G2	Stage III	T1	N1	MX	Negative	15.22825	High	Testing
TCGA-QK-A652	645	0	60	Male	G2	Stage III	T1	N1	MX	-	5.495974	High	Testing
TCGA-BB-8601	624	0	84	Male	G2	Stage III	T3	N1	MX	-	12.26883	High	Testing
TCGA-CV-6003	1665	0	50	Female	G2	Stage III	T2	N1	-	-	6.739576	High	Testing
TCGA-CV-6940	804	1	80	Female	G2	Stage III	T2	N1	-	-	14.65332	High	Testing

TCGA-CV-7410	6417	1	61	Male	GX	Stage III	T2	N1	-	-	4.241203	High	Testing
TCGA-CV-5444	2437	0	64	Male	G3	Stage IVA	T4a	N1	-	-	3.937022	High	Testing
TCGA-D6-A4Z9	539	0	59	Male	G2	Stage IVA	T2	N2	M0	-	3.520481	High	Testing
TCGA-CN-5374	1732	1	56	Female	G3	Stage IVA	T2	N2	M0	Positive	3.228554	High	Testing
TCGA-CV-6945	366	1	41	Male	G2	Stage IVA	T4a	N2	-	-	5.097959	High	Testing
TCGA-KU-A6H7	586	0	55	Female	G2	Stage IVA	T2	N2a	M0	Positive	5.495974	High	Testing
TCGA-CR-6474	564	1	51	Male	G2	Stage IVA	T2	N2b	M0	-	4.672831	High	Testing
TCGA-F7-A61W	14	0	51	Male	G2	Stage IVA	T2	N2b	M0	-	11.66027	High	Testing
TCGA-UF-A7JC	546	1	42	Male	G1	Stage IVA	T3	N2b	M0	-	3.520481	High	Testing
TCGA-CN-5367	352	1	60	Female	G2	Stage IVA	T4a	N2b	M0	-	5.428302	High	Testing
TCGA-UF-A7JV	90	1	62	Female	G2	Stage IVA	T4a	N2b	M0	-	4.107235	High	Testing
TCGA-H7-8502	458	0	50	Male	G2	Stage IVA	T4a	N2b	M0	-	21.49116	High	Testing
TCGA-CN-5359	377	1	59	Male	G2	Stage IVA	T4a	N2b	M0	Negative	8.61481	High	Testing
TCGA-UF-A7JK	424	1	59	Male	G2	Stage IVA	T4a	N2b	M0	-	5.711611	High	Testing
TCGA-CN-4742	397	1	48	Female	G3	Stage IVA	T4a	N2b	M0	Negative	5.495974	High	Testing
TCGA-IQ-A61O	421	1	43	Male	GX	Stage IVA	T2	N2b	MX	-	7.352734	High	Testing
TCGA-CN-A63U	964	0	50	Male	G3	Stage IVA	T3	N2b	MX	Negative	3.047446	High	Testing
TCGA-MZ-A7D7	547	0	51	Male	-	Stage IVA	T3	N2b	MX	Negative	38.47329	High	Testing
TCGA-HD-A4C1	11	0	41	Female	G1	Stage IVA	T4a	N2b	MX	Negative	4.029127	High	Testing
TCGA-BB-A6UO	268	1	61	Female	G2	Stage IVA	T4a	N2b	MX	-	3.148558	High	Testing
TCGA-CN-4737	625	0	19	Male	G2	Stage IVA	T2	N2b	-	Negative	4.02356	High	Testing
TCGA-CV-5979	1315	0	26	Male	G2	Stage IVA	T2	N2b	-	-	4.525229	High	Testing
TCGA-BB-4224	278	0	52	Male	G2	Stage IVA	T2	N2b	-	-	3.067113	High	Testing
TCGA-CQ-6224	1721	0	52	Male	G3	Stage IVA	T2	N2b	-	-	4.672831	High	Testing
TCGA-CQ-6218	1253	0	52	Female	G2	Stage IVA	T3	N2b	-	-	21.13434	High	Testing
TCGA-CV-5436	584	1	65	Male	G2	Stage IVA	T3	N2b	-	-	26.67514	High	Testing

TCGA-CV-5977	1840	0	66	Male	G2	Stage IVA	T3	N2b	-	-	15.22825	High	Testing
TCGA-CN-4726	142	1	68	Male	G2	Stage IVA	T3	N2b	-	Negative	8.365259	High	Testing
TCGA-CQ-5330	1897	0	69	Female	G3	Stage IVA	T3	N2b	-	-	14.65332	High	Testing
TCGA-D6-6826	348	1	64	Female	G2	Stage IVA	T4a	N2b	-	-	3.241631	High	Testing
TCGA-BB-4227	134	0	66	Male	G2	Stage IVA	T4a	N2b	-	-	6.739576	High	Testing
TCGA-CV-5435	2319	1	57	Male	G3	Stage IVA	T4a	N2b	-	-	4.672831	High	Testing
TCGA-CV-5441	2886	0	58	Male	G3	Stage IVA	T4a	N2b	-	-	8.101023	High	Testing
TCGA-RS-A6TO	387	1	82	Female	G2	Stage IVA	T4	N2c	M0	Negative	21.13434	High	Testing
TCGA-BA-A6DD	173	1	44	Male	G2	Stage IVA	T4a	N2c	M0	Negative	7.926787	High	Testing
TCGA-CN-5366	360	1	51	Male	G2	Stage IVA	T4a	N2c	M0	-	8.365259	High	Testing
TCGA-CN-5364	493	1	55	Male	G2	Stage IVA	T4a	N2c	M0	-	4.738878	High	Testing
TCGA-BA-A6DJ	407	1	62	Male	G2	Stage IVA	T4a	N2c	M0	Negative	14.43005	High	Testing
TCGA-QK-A8Z7	392	0	59	Male	-	Stage IVA	T4a	N2c	M0	-	4.08108	High	Testing
TCGA-CN-A63W	377	1	48	Female	G2	Stage IVA	T4a	N2c	MX	Negative	6.739576	High	Testing
TCGA-BA-5149	806	1	47	Male	G2	Stage IVA	T3	N2c	-	-	38.47329	High	Testing
TCGA-CN-6989	980	1	64	Male	G2	Stage IVA	T3	N2c	-	Negative	4.672831	High	Testing
TCGA-CV-7236	144	1	77	Female	G3	Stage IVA	T3	N2c	-	-	4.893449	High	Testing
TCGA-CN-6997	988	1	66	Male	G3	Stage IVA	T3	N2c	-	Negative	6.739576	High	Testing
TCGA-CV-7248	521	1	63	Female	G2	Stage IVA	T4a	N2c	-	-	5.616779	High	Testing
TCGA-CN-5363	253	1	48	Male	G3	Stage IVB	T4a	N3	M0	-	26.67514	High	Testing
TCGA-F7-A624	378	0	73	Male	G3	Stage II	T2	NX	M0	-	14.19046	High	Testing
TCGA-P3-A5QA	2182	0	41	Male	G3	Stage I	T1	NX	MX	-	3.137531	High	Testing
TCGA-BA-A4IF	895	0	59	Male	G2	-	TX	NX	MX	Negative	4.615294	High	Testing
TCGA-CV-7438	194	1	87	Female	G3	Stage I	T1	NX	-	-	8.365259	High	Testing
TCGA-CV-7180	327	1	34	Male	G2	Stage II	T2	NX	-	-	6.739576	High	Testing
TCGA-CV-7440	675	1	38	Male	GX	Stage II	T2	NX	-	-	3.241631	High	Testing

TCGA-CV-6962	126	1	65	Male	G2	Stage IVA	T4a	NX	-	-	4.02356	High	Testing
TCGA-BA-6872	384	1	47	Male	G2	-	TX	NX	-	-	3.137531	High	Testing
TCGA-DQ-5630	1030	0	73	Male	G2	-	TX	NX	-	-	14.74723	High	Testing
TCGA-MT-A7BN	469	0	74	Male	G3	Stage IVA	-	-	-	-	8.930032	High	Testing
TCGA-T2-A6WX	209	1	73	Female	G1	-	-	-	-	-	8.237798	High	Testing
TCGA-CV-A6JD	182	1	82	Female	G3	-	-	-	-	-	3.318871	High	Testing
TCGA-CR-7391	913	0	36	Female	G1	Stage I	T1	N0	M0	Negative	0.669927	Low	Testing
TCGA-CV-A6K0	606	0	58	Male	G3	Stage I	T1	N0	M0	-	0.9352	Low	Testing
TCGA-F7-8489	658	0	48	Male	G1	Stage II	T2	N0	M0	-	1.702302	Low	Testing
TCGA-CQ-7069	1274	0	77	Female	G2	Stage II	T2	N0	M0	-	1.564323	Low	Testing
TCGA-CQ-7072	2359	0	51	Male	G3	Stage II	T2	N0	M0	-	2.329799	Low	Testing
TCGA-IQ-A6SG	579	0	61	Female	G2	Stage III	T3	N0	M0	-	2.215433	Low	Testing
TCGA-CV-A464	1722	0	48	Male	G2	Stage III	T3	N0	M0	-	2.329799	Low	Testing
TCGA-D6-A6EP	424	0	62	Male	G3	Stage III	T3	N0	M0	-	1.564323	Low	Testing
TCGA-UF-A7JJ	549	0	68	Male	G1	Stage IVA	T4a	N0	M0	-	1.674187	Low	Testing
TCGA-UF-A7JO	631	1	79	Female	G2	Stage IVA	T4a	N0	M0	-	2.847718	Low	Testing
TCGA-CN-5360	2169	0	68	Male	G2	Stage IVA	T4a	N0	M0	-	0.220504	Low	Testing
TCGA-QK-A8ZB	542	0	68	Male	G2	Stage IVA	T4a	N0	M0	-	2.329799	Low	Testing
TCGA-CN-4739	1394	1	71	Male	G2	Stage IVA	T4a	N0	M0	-	2.329427	Low	Testing
TCGA-CN-4741	2239	0	75	Male	G2	Stage IVA	T4a	N0	M0	-	1.483881	Low	Testing
TCGA-CN-5369	1	1	90	Female	G3	Stage IVA	T4a	N0	M0	-	1.782673	Low	Testing
TCGA-CV-A45Y	2703	1	61	Male	G3	Stage IVA	T4a	N0	M0	-	1.702454	Low	Testing
TCGA-UF-A71E	1504	1	63	Male	G3	Stage IVA	T4a	N0	M0	-	1.22413	Low	Testing
TCGA-MT-A67A	914	0	85	Female	G2	Stage I	T1	N0	MX	-	2.279327	Low	Testing
TCGA-T2-A6X2	987	0	82	Male	G1	Stage III	T3	N0	MX	Negative	2.039953	Low	Testing
TCGA-P3-A6T5	882	1	79	Female	G2	Stage IVA	T4a	N0	MX	-	2.800436	Low	Testing

TCGA-D6-6516	773	0	69	Male	G2	Stage I	T1	N0	-	-	0.902189	Low	Testing
TCGA-D6-6825	491	0	73	Male	G2	Stage I	T1	N0	-	-	2.388399	Low	Testing
TCGA-D6-6827	568	0	55	Female	G3	Stage I	T1	N0	-	-	0.509972	Low	Testing
TCGA-CN-4722	1483	0	61	Female	G1	Stage II	T2	N0	-	-	0.669927	Low	Testing
TCGA-CR-7398	156	0	53	Female	G2	Stage II	T2	N0	-	-	1.012466	Low	Testing
TCGA-CV-7242	1095	0	60	Female	G2	Stage II	T2	N0	-	-	1.264058	Low	Testing
TCGA-CR-6488	379	0	68	Female	G2	Stage II	T2	N0	-	-	1.564323	Low	Testing
TCGA-CN-5358	261	1	60	Male	G2	Stage II	T2	N0	-	-	1.509103	Low	Testing
TCGA-BA-5556	725	0	58	Female	G3	Stage II	T2	N0	-	-	0.902189	Low	Testing
TCGA-D6-6515	403	1	82	Female	G3	Stage II	T2	N0	-	-	1.467802	Low	Testing
TCGA-CV-6433	641	0	57	Male	G3	Stage II	T2	N0	-	-	1.696357	Low	Testing
TCGA-CV-6961	76	1	61	Male	G3	Stage II	T2	N0	-	-	1.852617	Low	Testing
TCGA-CN-5356	1409	0	56	Male	G2	Stage III	T3	N0	-	-	2.405408	Low	Testing
TCGA-CV-6441	292	1	60	Male	G3	Stage III	T3	N0	-	-	0.902189	Low	Testing
TCGA-CN-6019	1038	0	61	Male	G2	Stage IVA	T4a	N0	-	Negative	1.564323	Low	Testing
TCGA-CN-6011	933	0	57	Male	G3	Stage IVA	T4a	N0	-	-	1.148783	Low	Testing
TCGA-QK-A6IJ	387	0	71	Male	G3	Stage III	T2	N1	M0	Negative	0.752415	Low	Testing
TCGA-CR-7371	94	1	45	Female	G2	Stage III	T3	N1	M0	-	1.363484	Low	Testing
TCGA-QK-A6VC	600	0	62	Female	G2	Stage IVA	T4a	N1	M0	Negative	1.702454	Low	Testing
TCGA-F7-7848	1131	0	47	Male	G2	Stage IVA	T4a	N1	M0	-	2.493424	Low	Testing
TCGA-CN-A497	1065	0	63	Male	G2	Stage III	T1	N1	MX	Negative	0.244053	Low	Testing
TCGA-P3-A6SX	1430	1	67	Male	G2	Stage III	T2	N1	MX	-	1.717504	Low	Testing
TCGA-P3-A6T4	62	1	54	Male	G2	Stage IVA	T4a	N1	MX	-	2.362729	Low	Testing
TCGA-HD-8314	670	0	58	Male	G4	Stage III	T1	N1	-	Positive	0.73984	Low	Testing
TCGA-CV-7243	954	0	50	Male	G2	Stage III	T2	N1	-	-	0.669927	Low	Testing
TCGA-CN-6020	205	1	58	Male	G2	Stage III	T2	N1	-	Negative	0.908349	Low	Testing

TCGA-CV-6956	217	1	67	Male	G2	Stage III	T3	N1	-	-	2.030684	Low	Testing
TCGA-CV-7422	1037	1	60	Female	G3	Stage IVA	T4a	N1	-	-	0.306804	Low	Testing
TCGA-BB-A6UM	393	0	52	Male	GX	Stage III	T1	N2	MX	Positive	0.586164	Low	Testing
TCGA-P3-A6T8	400	0	54	Male	G3	Stage IVA	T4a	N2	MX	-	2.740205	Low	Testing
TCGA-CV-A468	464	1	42	Male	G2	Stage IVA	T4a	N2a	M0	-	0.787939	Low	Testing
TCGA-IQ-A61I	2	1	63	Male	G3	Stage IVA	T1	N2b	M0	-	1.04267	Low	Testing
TCGA-BB-4223	3221	0	48	Male	G3	Stage IVA	T2	N2b	M0	-	0.831809	Low	Testing
TCGA-TN-A7HI	412	0	56	Male	G3	Stage IVA	T2	N2b	M0	Positive	0.462825	Low	Testing
TCGA-CX-A4AQ	1555	0	56	Male	G3	Stage IVA	T2	N2b	M0	-	1.23754	Low	Testing
TCGA-CR-7392	1425	0	67	Female	G1	Stage IVA	T3	N2b	M0	Negative	1.875713	Low	Testing
TCGA-CR-7397	754	0	44	Male	G2	Stage IVA	T3	N2b	M0	-	0.9352	Low	Testing
TCGA-TN-A7HL	619	0	59	Male	G2	Stage IVA	T3	N2b	M0	Positive	0.406412	Low	Testing
TCGA-QK-A8Z9	449	1	56	Male	G2	Stage IVA	T4a	N2b	M0	-	2.329799	Low	Testing
TCGA-UF-A7JS	680	1	59	Male	G2	Stage IVA	T4a	N2b	M0	-	1.317996	Low	Testing
TCGA-CR-6491	693	0	60	Male	G2	Stage IVA	T4a	N2b	M0	Negative	1.408724	Low	Testing
TCGA-CN-A6V6	635	0	59	Male	GX	Stage IVA	T2	N2b	MX	Positive	0.31803	Low	Testing
TCGA-P3-A5QF	330	1	49	Male	G2	Stage IVA	T4	N2b	MX	-	1.175658	Low	Testing
TCGA-CN-A641	367	0	47	Male	G2	Stage IVA	T4a	N2b	MX	Positive	0.565473	Low	Testing
TCGA-HD-7754	783	0	69	Male	G1	Stage IVA	T2	N2b	-	-	0.815133	Low	Testing
TCGA-CQ-6222	2016	0	63	Male	G2	Stage IVA	T2	N2b	-	-	0.449817	Low	Testing
TCGA-CV-7446	1093	1	66	Male	G2	Stage IVA	T2	N2b	-	-	2.247559	Low	Testing
TCGA-CV-7424	453	1	67	Male	G2	Stage IVA	T3	N2b	-	-	1.509103	Low	Testing
TCGA-CQ-5334	129	1	87	Male	G2	Stage IVA	T3	N2b	-	-	1.214005	Low	Testing
TCGA-CN-4735	1737	0	52	Male	G3	Stage IVA	T3	N2b	-	Negative	1.363484	Low	Testing
TCGA-T2-A6WZ	484	1	53	Male	G2	-	T3	N2b	-	Negative	1.0852	Low	Testing
TCGA-CV-5434	3314	1	60	Male	G2	Stage IVA	T4a	N2b	-	-	0.70599	Low	Testing

TCGA-CV-5442	2327	0	76	Female	G3	Stage IVA	T4a	N2b	-	-	2.173301	Low	Testing
TCGA-CN-6988	318	0	47	Male	G3	Stage IVA	T4a	N2b	-	Negative	1.509103	Low	Testing
TCGA-CV-7433	601	1	49	Male	G3	Stage IVA	T4a	N2b	-	-	2.747196	Low	Testing
TCGA-CV-5440	3270	0	52	Male	G3	Stage IVA	T4a	N2b	-	-	1.120597	Low	Testing
TCGA-CV-6939	666	1	60	Male	G3	Stage IVA	T4a	N2b	-	-	1.175658	Low	Testing
TCGA-CN-6023	1584	0	73	Male	GX	Stage IVA	T3	N2c	M0	Negative	1.017689	Low	Testing
TCGA-BA-A6D8	850	0	59	Male	G2	Stage IVA	T4a	N2c	M0	Negative	1.509103	Low	Testing
TCGA-CN-6024	337	1	66	Male	G2	Stage IVA	T4a	N2c	M0	Negative	2.262906	Low	Testing
TCGA-BB-7862	1117	0	67	Male	G3	Stage IVA	T3	N2c	-	-	2.329799	Low	Testing
TCGA-CV-7415	695	1	60	Male	G2	Stage IVA	T4a	N2c	-	-	1.509103	Low	Testing
TCGA-CV-6936	166	1	68	Male	G2	Stage IVA	T4a	N2c	-	-	1.61509	Low	Testing
TCGA-CV-7089	1972	1	74	Male	G2	Stage IVA	T4a	N2c	-	-	2.455213	Low	Testing
TCGA-BA-A8YP	499	0	50	Male	G2	Stage IVB	T4a	N3	M0	Negative	2.301112	Low	Testing
TCGA-QK-A6V9	833	0	56	Male	G2	Stage II	T2	NX	M0	Positive	0.195478	Low	Testing
TCGA-P3-A6T6	395	1	53	Male	G3	Stage IVA	T4a	NX	MX	-	0.818854	Low	Testing
TCGA-CN-A49C	645	0	67	Male	GX	-	TX	NX	MX	Positive	0.368839	Low	Testing
TCGA-CV-7101	160	1	80	Male	G2	Stage II	T2	NX	-	-	0.9352	Low	Testing
TCGA-BA-5153	1762	1	51	Male	G2	-	T2	NX	-	-	0.386253	Low	Testing
TCGA-CV-6943	602	1	74	Male	G2	Stage III	T3	NX	-	-	0.9352	Low	Testing
TCGA-CV-6954	2002	1	59	Male	G2	Stage IVA	T4a	NX	-	-	1.509103	Low	Testing
TCGA-BA-6870	451	1	60	Female	G2	-	TX	NX	-	-	2.301112	Low	Testing
TCGA-CN-6021	276	1	63	Female	G2	-	TX	NX	-	-	1.34366	Low	Testing
TCGA-CR-7374	30	0	67	Female	G2	-	TX	NX	-	-	2.030684	Low	Testing
TCGA-CR-7388	823	1	70	Female	G2	-	TX	NX	-	-	2.847718	Low	Testing
TCGA-CR-7370	105	0	72	Female	G2	-	TX	NX	-	-	0.9352	Low	Testing
TCGA-CR-7404	1472	0	53	Male	G2	-	TX	NX	-	Positive	0.21272	Low	Testing

TCGA-CR-7389	392	0	55	Male	G2	-	TX	NX	-	-	0.9352	Low	Testing
TCGA-BA-6871	108	1	75	Male	G2	-	TX	NX	-	-	2.744569	Low	Testing
TCGA-DQ-7595	1190	0	53	Male	G3	-	TX	NX	-	Positive	2.186048	Low	Testing
TCGA-CR-5248	1663	0	53	Male	G3	-	TX	NX	-	-	0.195478	Low	Testing
TCGA-CR-5250	799	0	71	Male	G3	-	TX	NX	-	Positive	0.195478	Low	Testing
TCGA-DQ-7594	1218	0	47	Male	G4	-	TX	NX	-	Positive	0.815133	Low	Testing
TCGA-DQ-7590	1413	0	51	Male	G4	-	TX	NX	-	Positive	1.176155	Low	Testing
TCGA-DQ-7593	1224	0	58	Male	G4	-	TX	NX	-	Positive	2.482109	Low	Testing
TCGA-DQ-7589	1409	0	70	Male	G4	-	TX	NX	-	Negative	0.513195	Low	Testing
TCGA-BB-7866	1368	0	40	Male	GX	-	TX	NX	-	Positive	0.949996	Low	Testing
TCGA-T2-A6X0	216	0	49	Male	G2	-	TX	-	-	Positive	1.346696	Low	Testing
TCGA-CV-A6JN	906	0	53	Male	G1	-	-	-	-	-	1.564323	Low	Testing
TCGA-CR-6477	514	0	56	Female	G2	-	-	-	-	Negative	1.190818	Low	Testing
TCGA-CV-A461	2064	1	65	Male	G2	-	-	-	-	-	1.545061	Low	Testing
TCGA-MZ-A6I9	489	1	68	Male	G2	-	-	-	-	Positive	0.406412	Low	Testing
TCGA-CV-A6JE	1075	0	78	Male	G2	-	-	-	-	-	1.346966	Low	Testing
TCGA-CR-6482	345	0	62	Male	G3	-	-	-	-	Positive	0.605284	Low	Testing
TCGA-CR-6473	1125	0	68	Male	G3	-	-	-	-	-	0.934228	Low	Testing
TCGA-DQ-7596	1265	0	48	Male	G4	-	-	-	-	-	1.175658	Low	Testing
TCGA-BA-A6DL	623	0	59	Male	G2	-	T0	N0	M0	Negative	3.241631	High	Training
TCGA-BA-A6DB	216	0	24	Female	G1	Stage I	T1	N0	M0	Negative	4.672831	High	Training
TCGA-MT-A51W	437	0	52	Female	G2	Stage I	T1	N0	M0	Negative	7.829185	High	Training
TCGA-CR-7383	521	1	79	Female	G2	Stage I	T1	N0	M0	-	8.506496	High	Training
TCGA-CV-A45T	4856	1	64	Female	G3	-	T1	N0	M0	-	3.137531	High	Training
TCGA-BA-A6DE	440	0	70	Female	G2	Stage II	T2	N0	M0	Negative	4.525229	High	Training
TCGA-CQ-A4CE	897	0	76	Female	G2	Stage II	T2	N0	M0	-	3.469295	High	Training

TCGA-D6-8569	770	0	52	Male	G2	Stage II	T2	N0	M0	-	3.696689	High	Training
TCGA-MT-A67D	56	0	55	Male	G2	Stage II	T2	N0	M0	-	8.101023	High	Training
TCGA-CQ-A4CH	379	1	58	Male	G2	Stage II	T2	N0	M0	-	8.365259	High	Training
TCGA-CV-A45W	1398	1	75	Male	G2	Stage II	T2	N0	M0	-	5.711611	High	Training
TCGA-CV-A465	215	1	24	Male	G1	Stage III	T3	N0	M0	-	21.13434	High	Training
TCGA-F7-A61S	576	0	62	Male	G1	Stage III	T3	N0	M0	-	5.495974	High	Training
TCGA-CQ-A4CG	430	1	78	Female	G2	Stage III	T3	N0	M0	-	14.90073	High	Training
TCGA-BA-4075	283	1	49	Male	G2	Stage III	T3	N0	M0	-	38.47329	High	Training
TCGA-D6-A4ZB	376	0	61	Male	G2	Stage III	T3	N0	M0	-	5.097959	High	Training
TCGA-CV-A45V	32	1	87	Female	G1	Stage IVA	T4	N0	M0	-	6.112745	High	Training
TCGA-CV-A6K2	317	1	79	Male	G1	Stage IVA	T4	N0	M0	-	4.525229	High	Training
TCGA-UF-A7JH	896	0	59	Male	G1	Stage IVA	T4a	N0	M0	-	4.539068	High	Training
TCGA-CN-4740	839	1	79	Female	G2	Stage IVA	T4a	N0	M0	-	8.365259	High	Training
TCGA-D6-A6EO	759	0	44	Male	G2	Stage IVA	T4a	N0	M0	-	6.54607	High	Training
TCGA-D6-A6ES	389	0	50	Male	G2	Stage IVA	T4a	N0	M0	-	4.73233	High	Training
TCGA-UF-A7JT	993	1	72	Female	G3	Stage IVA	T4a	N0	M0	-	4.92481	High	Training
TCGA-HD-8634	385	1	51	Female	G2	Stage I	T1	N0	MX	-	5.00101	High	Training
TCGA-MT-A67F	384	0	60	Female	G2	Stage IVA	T4	N0	MX	-	12.26883	High	Training
TCGA-CQ-5325	654	1	65	Male	G2	Stage I	T1	N0	-	-	5.616779	High	Training
TCGA-BB-7872	1168	0	63	Male	G3	Stage I	T1	N0	-	Positive	5.495974	High	Training
TCGA-HD-7917	836	1	62	Male	G1	Stage II	T2	N0	-	-	12.26883	High	Training
TCGA-CQ-5329	2143	0	46	Female	G2	Stage II	T2	N0	-	-	3.241631	High	Training
TCGA-CQ-7065	1628	0	40	Male	G2	Stage II	T2	N0	-	-	3.137531	High	Training
TCGA-CN-4725	1157	0	60	Male	G2	Stage II	T2	N0	-	Negative	5.678326	High	Training
TCGA-CQ-6229	1179	0	61	Male	G2	Stage II	T2	N0	-	-	4.73233	High	Training
TCGA-CN-4734	1690	0	70	Male	G2	Stage II	T2	N0	-	Negative	6.739576	High	Training

TCGA-BB-7863	1025	0	43	Female	G2	Stage III	T3	N0	-	-	3.357883	High	Training
TCGA-CV-6959	256	1	48	Male	G2	Stage III	T3	N0	-	-	6.739576	High	Training
TCGA-D6-6517	292	0	59	Male	G2	Stage III	T3	N0	-	-	6.656592	High	Training
TCGA-CV-5432	3930	0	68	Male	G3	Stage III	T3	N0	-	-	5.495974	High	Training
TCGA-HD-7832	836	0	52	Male	G2	Stage IVA	T4a	N0	-	-	9.339469	High	Training
TCGA-CV-7261	1512	0	57	Male	G2	Stage IVA	T4a	N0	-	-	3.656632	High	Training
TCGA-CN-6995	112	1	78	Male	G2	Stage IVA	T4a	N0	-	-	7.829185	High	Training
TCGA-BB-7870	2016	0	58	Male	G3	Stage IVA	T4a	N0	-	-	4.843041	High	Training
TCGA-CQ-A4CB	893	0	59	Male	G2	Stage III	T1	N1	M0	-	5.331697	High	Training
TCGA-C9-A47Z	191	1	72	Female	G1	Stage III	T2	N1	M0	-	8.365259	High	Training
TCGA-QK-A6IG	222	1	69	Male	G2	Stage III	T2	N1	M0	-	8.237798	High	Training
TCGA-D6-A6EN	687	0	71	Male	G3	Stage III	T2	N1	M0	-	11.66027	High	Training
TCGA-BA-A4IH	622	0	57	Male	G3	-	T2	N1	M0	Positive	3.696689	High	Training
TCGA-CN-5370	259	1	78	Male	G3	Stage III	T3	N1	M0	Negative	21.13434	High	Training
TCGA-CQ-A4C7	353	1	88	Male	G3	Stage III	T3	N1	M0	-	3.937022	High	Training
TCGA-H7-8501	461	0	54	Male	GX	Stage IVA	T4a	N1	M0	-	21.96355	High	Training
TCGA-KU-A66S	406	1	69	Female	G2	Stage III	T1	N1	MX	-	21.96355	High	Training
TCGA-HD-A6HZ	111	0	79	Female	G2	Stage III	T2	N1	MX	-	3.962253	High	Training
TCGA-CN-A49B	904	0	71	Male	G3	Stage III	T3	N1	MX	Negative	3.57992	High	Training
TCGA-BA-7269	1273	0	61	Male	G1	Stage III	T2	N1	-	-	8.930032	High	Training
TCGA-CX-7086	573	0	53	Male	G2	Stage III	T3	N1	-	-	3.357883	High	Training
TCGA-P3-A6T3	577	1	49	Male	G2	Stage IVA	T3	N2	MX	-	10.95068	High	Training
TCGA-CV-7102	56	1	76	Female	G3	Stage IVA	T3	N2	-	-	2.892772	High	Training
TCGA-CQ-6219	479	1	50	Female	G2	Stage IVA	T3	N2a	-	-	12.26883	High	Training
TCGA-CV-5430	4241	0	61	Male	G3	Stage IVA	T4a	N2a	-	-	4.539068	High	Training
TCGA-MT-A51X	242	0	30	Male	G1	Stage IVA	T1	N2b	M0	-	15.22825	High	Training

TCGA-F7-A623	616	0	70	Male	G1	Stage IVA	T1	N2b	M0	-	5.150199	High	Training
TCGA-IQ-A61J	1021	0	54	Male	G1	Stage IVA	T2	N2b	M0	-	5.616779	High	Training
TCGA-H7-A6C4	414	0	35	Female	G2	Stage IVA	T2	N2b	M0	Negative	8.101023	High	Training
TCGA-CV-A45X	198	1	47	Male	G2	Stage IVA	T2	N2b	M0	-	4.213524	High	Training
TCGA-KU-A6H8	327	1	41	Male	G3	Stage IVA	T2	N2b	M0	-	6.112745	High	Training
TCGA-CN-A6V3	742	0	61	Male	G3	Stage IVA	T3	N2b	M0	-	10.00496	High	Training
TCGA-BB-A5HY	321	1	64	Male	G3	Stage IVA	T3	N2b	M0	Negative	38.47329	High	Training
TCGA-CV-A45U	1079	1	59	Male	G1	Stage IVA	T4	N2b	M0	-	5.711611	High	Training
TCGA-BB-A5HU	782	0	47	Male	G2	Stage IVA	T4	N2b	M0	Negative	15.22825	High	Training
TCGA-UF-A7JD	739	1	71	Male	G3	Stage IVA	T4	N2b	M0	-	5.616779	High	Training
TCGA-QK-A6IH	653	0	65	Female	G2	Stage IVB	T4b	N2b	M0	-	4.039041	High	Training
TCGA-QK-A8ZA	371	1	60	Male	G2	-	T2	N2b	MX	Positive	10.16529	High	Training
TCGA-P3-A6T7	487	1	55	Male	G2	Stage IVA	T3	N2b	MX	-	12.87969	High	Training
TCGA-QK-AA3K	253	0	60	Male	G2	Stage IVA	T3	N2b	MX	Positive	2.982167	High	Training
TCGA-HD-A634	130	1	56	Male	G3	Stage IVA	T3	N2b	MX	Positive	15.22825	High	Training
TCGA-CN-A63T	225	0	60	Male	G3	Stage IVA	T3	N2b	MX	Negative	26.67514	High	Training
TCGA-P3-A5Q6	480	1	49	Male	G3	Stage IVA	T4a	N2b	MX	-	3.098898	High	Training
TCGA-CV-7104	393	1	61	Female	G2	Stage IVA	T2	N2b	-	-	8.365259	High	Training
TCGA-CV-6952	185	1	65	Female	G1	Stage IVA	T3	N2b	-	-	21.13434	High	Training
TCGA-CN-6996	530	1	58	Female	G2	Stage IVA	T3	N2b	-	-	3.241631	High	Training
TCGA-CN-6998	357	1	53	Male	G2	Stage IVA	T3	N2b	-	Negative	7.212905	High	Training
TCGA-CN-6017	853	1	55	Male	G2	Stage IVA	T3	N2b	-	Negative	3.357883	High	Training
TCGA-CN-6992	1066	0	61	Male	G2	Stage IVA	T3	N2b	-	Negative	4.672831	High	Training
TCGA-CV-5439	546	1	62	Male	G2	Stage IVA	T3	N2b	-	-	5.608409	High	Training
TCGA-CR-6493	282	1	69	Male	G2	Stage IVA	T3	N2b	-	-	4.672831	High	Training
TCGA-BA-4074	462	1	69	Male	G3	Stage IVA	T2	N2c	M0	-	25.83254	High	Training

TCGA-BA-A6DA	351	0	41	Female	G2	Stage IVA	T4a	N2c	M0	Positive	5.886101	High	Training
TCGA-QK-A6II	284	1	52	Male	G2	Stage IVA	T4a	N2c	M0	-	5.717099	High	Training
TCGA-BB-4217	187	0	68	Male	G3	Stage IVA	T4a	N2c	M0	-	11.80563	High	Training
TCGA-4P-AA8J	102	0	66	Male	G2	Stage IVA	T2	N2c	MX	Negative	7.926787	High	Training
TCGA-T3-A92N	95	1	79	Male	G3	Stage IVA	T2	N2c	MX	-	18.50505	High	Training
TCGA-IQ-A61G	360	0	57	Male	G2	Stage IVA	T4a	N2c	MX	-	8.237798	High	Training
TCGA-CV-6935	295	1	67	Male	G3	Stage IVA	T3	N2c	-	-	5.097959	High	Training
TCGA-CV-5431	522	1	73	Male	G3	Stage IVA	T3	N2c	-	-	8.506496	High	Training
TCGA-CX-7219	1045	0	47	Male	G2	Stage IVA	T4a	N2c	-	-	4.539068	High	Training
TCGA-CV-6951	915	1	57	Male	G2	Stage IVA	T4a	N2c	-	-	4.672831	High	Training
TCGA-CN-A642	82	1	57	Male	G3	Stage IVB	T4a	N3	M0	Negative	12.26883	High	Training
TCGA-CN-5365	351	1	38	Male	G2	Stage IVB	T2	N3	-	-	25.83254	High	Training
TCGA-CV-5978	215	1	53	Female	G2	Stage IVB	T4a	N3	-	-	3.285663	High	Training
TCGA-CV-6948	1289	1	79	Female	G2	Stage IVB	T4a	N3	-	-	4.11943	High	Training
TCGA-F7-A622	359	1	75	Male	G1	Stage III	T3	NX	M0	-	4.738878	High	Training
TCGA-BA-A6DI	336	1	62	Male	G2	-	TX	NX	M0	Negative	4.672831	High	Training
TCGA-QK-A64Z	641	1	79	Female	G1	Stage IVA	T4	NX	MX	-	7.926787	High	Training
TCGA-BA-A6DG	69	1	49	Male	G2	-	TX	NX	MX	Negative	7.305912	High	Training
TCGA-CN-4736	395	1	70	Female	G2	-	T1	NX	-	-	4.81223	High	Training
TCGA-DQ-5625	1133	1	52	Female	G2	-	TX	NX	-	-	4.3677	High	Training
TCGA-BA-4076	415	1	39	Male	G2	-	TX	NX	-	-	12.26883	High	Training
TCGA-CR-5247	358	0	48	Male	G2	-	TX	NX	-	-	4.525229	High	Training
TCGA-DQ-7592	1143	0	57	Male	G2	-	TX	NX	-	Negative	5.495974	High	Training
TCGA-DQ-5629	941	1	64	Male	G2	-	TX	NX	-	Negative	8.365259	High	Training
TCGA-DQ-5631	548	1	52	Male	G3	-	TX	NX	-	-	4.02356	High	Training
TCGA-BA-4078	276	1	83	Male	G2	-	-	-	-	-	3.444764	High	Training

TCGA-CR-6478	183	1	66	Female	G3	-	-	-	-	-	8.237798	High	Training
TCGA-CR-7372	759	0	45	Male	G1	Stage I	T1	N0	M0	Negative	1.301217	Low	Training
TCGA-CV-A45P	639	0	82	Female	G2	Stage I	T1	N0	M0	-	2.186048	Low	Training
TCGA-CR-7401	1077	0	64	Male	G2	Stage I	T1	N0	M0	-	1.392826	Low	Training
TCGA-CR-6487	234	0	50	Male	G3	Stage I	T1	N0	M0	Positive	0.21272	Low	Training
TCGA-QK-AA3J	466	0	69	Male	G3	Stage I	T1	N0	M0	-	0.9352	Low	Training
TCGA-UF-A719	1663	0	54	Male	G1	Stage II	T2	N0	M0	-	0.949996	Low	Training
TCGA-CR-6470	1521	0	38	Male	G2	Stage II	T2	N0	M0	-	0.220504	Low	Training
TCGA-CV-A6JT	852	0	65	Male	G2	Stage II	T2	N0	M0	-	0.752415	Low	Training
TCGA-RS-A6TP	516	0	58	Male	G3	Stage II	T2	N0	M0	Positive	0.195478	Low	Training
TCGA-CR-5249	1152	0	35	Female	GX	Stage II	T2	N0	M0	-	0.220504	Low	Training
TCGA-C9-A480	386	0	45	Female	G1	Stage III	T3	N0	M0	-	2.247559	Low	Training
TCGA-CV-A6JY	646	0	69	Male	G1	Stage IVA	T4a	N0	M0	-	0.902189	Low	Training
TCGA-BA-4077	1134	1	45	Female	G2	Stage IVA	T4a	N0	M0	-	1.23754	Low	Training
TCGA-UF-A71D	1461	0	54	Female	G2	Stage IVA	T4a	N0	M0	-	1.61509	Low	Training
TCGA-CV-A6K1	685	0	65	Male	G2	Stage IVA	T4a	N0	M0	-	2.279327	Low	Training
TCGA-CR-7394	1346	0	70	Male	G2	Stage IVA	T4a	N0	M0	Negative	0.902189	Low	Training
TCGA-UF-A7J9	1358	0	75	Male	G2	Stage IVA	T4a	N0	M0	-	2.256207	Low	Training
TCGA-D6-A74Q	710	0	67	Male	G3	Stage IVA	T4a	N0	M0	-	1.564323	Low	Training
TCGA-CV-A6JU	110	0	61	Female	G2	Stage IVB	T4b	N0	M0	-	0.752415	Low	Training
TCGA-CN-A499	717	0	60	Female	G2	Stage II	T2	N0	MX	Positive	2.059386	Low	Training
TCGA-IQ-A61E	1147	0	55	Female	G2	Stage III	T3	N0	MX	-	2.176565	Low	Training
TCGA-P3-A6T0	578	0	47	Female	G2	Stage IVA	T4a	N0	MX	-	2.847718	Low	Training
TCGA-CN-A63V	679	0	59	Male	G2	Stage IVA	T4a	N0	MX	Negative	1.702454	Low	Training
TCGA-HD-A633	421	0	74	Male	G2	Stage IVA	T4a	N0	MX	-	1.721886	Low	Training
TCGA-CN-5373	1584	0	55	Female	G1	Stage I	T1	N0	-	Negative	1.821857	Low	Training

TCGA-CX-7085	321	0	77	Female	G2	Stage I	T1	N0	-	-	0.449817	Low	Training
TCGA-HD-7753	866	0	62	Male	G2	Stage I	T1	N0	-	-	1.363484	Low	Training
TCGA-CQ-7067	509	0	75	Female	G3	Stage I	T1	N0	-	-	1.875713	Low	Training
TCGA-IQ-7631	1172	0	60	Female	G1	Stage II	T2	N0	-	-	0.244053	Low	Training
TCGA-CV-7235	2347	0	67	Male	G3	Stage II	T2	N0	-	-	1.327116	Low	Training
TCGA-WA-A7H4	443	0	69	Male	G3	Stage II	T2	N0	-	-	2.329799	Low	Training
TCGA-CQ-5333	341	1	74	Male	G3	Stage II	T2	N0	-	-	2.847718	Low	Training
TCGA-CV-7406	1748	1	49	Male	GX	Stage II	T2	N0	-	-	1.483881	Low	Training
TCGA-CV-6953	1641	1	80	Female	G1	Stage III	T3	N0	-	-	1.34366	Low	Training
TCGA-BA-6869	644	0	62	Male	G2	Stage III	T3	N0	-	-	1.702302	Low	Training
TCGA-CV-5443	2784	0	63	Male	G3	Stage III	T3	N0	-	-	0.815133	Low	Training
TCGA-CV-6436	1899	0	62	Male	G1	Stage IVA	T4a	N0	-	-	0.588788	Low	Training
TCGA-D6-6824	77	0	61	Male	G2	Stage IVA	T4a	N0	-	-	0.755693	Low	Training
TCGA-CR-7393	993	0	26	Male	G2	Stage III	T1	N1	M0	Negative	0.669927	Low	Training
TCGA-CV-A45R	5480	0	46	Male	G1	Stage III	T2	N1	M0	-	0.449817	Low	Training
TCGA-IQ-A6SH	471	0	55	Male	G1	Stage III	T2	N1	M0	-	2.329799	Low	Training
TCGA-D6-A6EK	875	0	67	Male	G1	Stage III	T2	N1	M0	-	1.564323	Low	Training
TCGA-CQ-A4C9	707	0	56	Male	G2	Stage III	T2	N1	M0	-	1.34366	Low	Training
TCGA-CR-6467	1777	0	59	Male	G3	Stage III	T2	N1	M0	-	0.392066	Low	Training
TCGA-CR-6492	479	0	78	Male	G3	Stage III	T2	N1	M0	Negative	1.875713	Low	Training
TCGA-F7-A50G	616	0	66	Male	G1	Stage III	T3	N1	M0	-	1.317996	Low	Training
TCGA-CN-6022	281	1	49	Male	G3	Stage IVA	T4a	N1	M0	Negative	2.847718	Low	Training
TCGA-D6-A6EQ	368	0	57	Male	G3	Stage IVA	T4a	N1	M0	-	2.740205	Low	Training
TCGA-P3-A5QE	1559	0	49	Male	G2	Stage III	T2	N1	MX	-	0.73984	Low	Training
TCGA-HD-A6I0	210	0	56	Male	G1	Stage IVA	T4a	N1	MX	-	1.190818	Low	Training
TCGA-CN-4733	1586	0	61	Male	G3	Stage III	T1	N1	-	Negative	1.702302	Low	Training

TCGA-BB-7861	682	0	56	Male	-	Stage III	T1	N1	-	Positive	1.346696	Low	Training
TCGA-CV-6933	2741	1	53	Male	G2	Stage III	T3	N1	-	-	1.773525	Low	Training
TCGA-CV-5973	2641	0	62	Female	G3	Stage III	T3	N1	-	-	0.848741	Low	Training
TCGA-CN-6016	1443	0	64	Male	G2	Stage IVA	T4a	N1	-	Negative	0.449817	Low	Training
TCGA-CV-A460	1838	1	72	Male	G3	-	T1	N2	M0	-	1.467802	Low	Training
TCGA-P3-A5Q5	910	0	54	Male	G3	Stage IVA	T2	N2	MX	-	0.73984	Low	Training
TCGA-HD-7831	667	0	74	Male	G2	Stage IVA	T2	N2	-	-	1.392826	Low	Training
TCGA-CN-5361	2120	1	80	Male	G2	Stage IVA	T4a	N2a	M0	-	2.440897	Low	Training
TCGA-UF-A7JF	1686	0	80	Male	G2	Stage IVA	T4a	N2a	M0	-	0.588788	Low	Training
TCGA-CN-A6V1	603	0	59	Male	GX	Stage IVA	T2	N2a	MX	Positive	1.23754	Low	Training
TCGA-CV-5971	701	0	60	Male	G2	Stage IVA	T4a	N2a	-	-	1.509103	Low	Training
TCGA-TN-A7HJ	403	0	51	Male	G2	Stage IVA	T4a	N2b	M0	-	0.9352	Low	Training
TCGA-CN-A6V7	594	0	40	Male	GX	Stage IVA	T2	N2b	MX	Positive	0.671441	Low	Training
TCGA-P3-A6T2	2298	0	45	Male	G2	Stage IVA	T3	N2b	MX	-	1.942326	Low	Training
TCGA-CN-A6UY	713	0	57	Male	G2	Stage IVA	T3	N2b	MX	Positive	0.671441	Low	Training
TCGA-H7-A76A	637	0	57	Male	G2	-	TX	N2b	MX	Positive	1.702454	Low	Training
TCGA-CV-7103	1591	1	49	Male	G2	Stage IVA	T2	N2b	-	-	0.848741	Low	Training
TCGA-CV-6934	65	1	66	Female	G2	Stage IVA	T3	N2b	-	-	2.329799	Low	Training
TCGA-BA-6873	122	0	28	Male	G2	Stage IVA	T4a	N2b	-	-	2.535297	Low	Training
TCGA-CV-5976	1478	0	50	Male	G2	Stage IVA	T4a	N2b	-	-	2.535297	Low	Training
TCGA-CV-5970	406	1	59	Male	G2	Stage IVA	T4a	N2b	-	-	2.643478	Low	Training
TCGA-CV-7418	789	1	62	Male	G2	Stage IVA	T4a	N2b	-	-	2.455213	Low	Training
TCGA-CN-6013	727	1	56	Male	G3	Stage IVA	T4a	N2b	-	Negative	1.702454	Low	Training
TCGA-CR-7382	796	0	49	Male	G2	Stage IVA	T2	N2c	M0	-	1.875713	Low	Training
TCGA-CV-A6JO	197	1	69	Male	G2	Stage IVA	T3	N2c	M0	-	2.847718	Low	Training
TCGA-CR-7399	181	0	60	Female	G3	Stage IVA	T3	N2c	M0	-	1.408724	Low	Training

TCGA-HD-8224	446	1	63	Male	G3	Stage IVA	T3	N2c	M0	Negative	2.104652	Low	Training
TCGA-UF-A71A	86	1	67	Male	G1	Stage IVA	T4a	N2c	M0	-	2.455213	Low	Training
TCGA-T3-A92M	417	0	52	Male	G2	Stage IVA	T4a	N2c	MX	-	1.509103	Low	Training
TCGA-BB-7864	1527	0	61	Male	G2	Stage IVA	T2	N2c	-	-	2.030684	Low	Training
TCGA-CQ-5327	1660	0	61	Female	G2	Stage IVA	T3	N2c	-	-	1.467802	Low	Training
TCGA-BA-5555	520	0	54	Male	G2	Stage IVA	T3	N2c	-	-	1.34366	Low	Training
TCGA-CV-6950	459	1	64	Male	G2	Stage IVA	T3	N2c	-	-	1.96654	Low	Training
TCGA-CN-6012	1460	0	66	Male	G2	Stage IVA	T3	N2c	-	Negative	0.489492	Low	Training
TCGA-BB-7871	750	0	64	Female	G2	Stage IVA	T4a	N2c	-	-	0.607992	Low	Training
TCGA-CN-6010	1523	0	53	Male	G2	Stage IVA	T4a	N2c	-	Negative	1.509103	Low	Training
TCGA-P3-A6SW	1120	0	50	Male	G3	Stage IVB	T3	N3	MX	-	0.185782	Low	Training
TCGA-CQ-7063	2133	0	59	Female	G1	Stage I	T1	NX	M0	-	1.515678	Low	Training
TCGA-CR-7385	997	0	42	Male	G2	-	T1	NX	M0	-	0.44226	Low	Training
TCGA-BA-A4IG	855	0	77	Male	G3	-	T1	NX	MX	-	1.467802	Low	Training
TCGA-BB-8596	2161	0	69	Female	G3	Stage IVA	T4a	NX	MX	-	2.847718	Low	Training
TCGA-CV-7407	1081	1	67	Female	G2	Stage II	T2	NX	-	-	2.759165	Low	Training
TCGA-CV-7177	663	1	82	Female	G2	Stage II	T2	NX	-	-	1.317996	Low	Training
TCGA-CV-7437	506	1	77	Male	GX	Stage II	T2	NX	-	-	2.643478	Low	Training
TCGA-CQ-6221	1000	0	79	Male	G3	-	T2	NX	-	-	0.888812	Low	Training
TCGA-BB-4228	559	0	50	Male	GX	Stage III	T3	NX	-	Positive	2.00568	Low	Training
TCGA-DQ-5624	1778	0	43	Female	G2	-	TX	NX	-	-	2.006083	Low	Training
TCGA-BA-6868	472	1	53	Male	G2	-	TX	NX	-	-	2.847718	Low	Training
TCGA-CR-7402	911	0	68	Male	G2	-	TX	NX	-	-	0.368839	Low	Training
TCGA-CR-5243	2562	0	51	Male	G3	-	TX	NX	-	-	0.31803	Low	Training
TCGA-BA-5559	2083	1	71	Male	G3	-	TX	NX	-	-	0.21272	Low	Training
TCGA-BB-4225	146	0	73	Male	G3	-	TX	NX	-	-	0.35582	Low	Training

TCGA-DQ-7591	622	0	62	Male	G4	-	TX	NX	-	Positive	0.62334	Low	Training
TCGA-CV-A45Q	5152	1	69	Female	G1	-	-	-	-	-	1.317996	Low	Training
TCGA-CR-6481	311	0	47	Male	G2	-	-	-	-	Positive	0.605284	Low	Training
TCGA-CR-6480	362	0	53	Male	G2	-	-	-	-	Positive	0.44226	Low	Training
TCGA-CV-A45O	851	0	57	Male	G2	-	-	-	-	-	1.410561	Low	Training
TCGA-UP-A6WW	518	0	58	Male	G2	-	-	-	-	-	1.134638	Low	Training
TCGA-CR-6472	1050	0	59	Male	G3	-	-	-	-	-	0.106059	Low	Training
TCGA-QK-A6IF	704	0	61	Male	GX	-	-	-	-	Positive	0.326979	Low	Training
TCGA-MZ-A5BI	217	1	53	Male	-	-	-	-	-	Positive	1.702454	Low	Training

Abbreviations: HPV, human papilloma virus.

"-" indicates the absence of pertinent data in the TCGA database.

GX, TX, NX, MX: unable to evaluate.

Extended Data Table S2. A list of 42 potential therapeutic agents has been predicted based on drug sensitivity analysis.

Drug name	Drug sensitive group	*p*-value	Drug name	Drug sensitive group	*p*-value	Drug name	Drug sensitive group	*p*-value
Afuresertib	Low-risk	0.0001	AGI-5198	Low-risk	0.00043	Axitinib	Low-risk	0.00061
AZ6102	Low-risk	0.00018	AZD1208	Low-risk	1.3e-06	AZD6482	Low-risk	1.9e-05
BIBR-1532	Low-risk	7.5e-06	CDK9_5038	Low-risk	0.00087	Cediranib	Low-risk	0.00046
Cisplatin	High-risk	7.4e-05	Cyclophosphamide	Low-risk	0.00033	Dasatinib	High-risk	0.00015
Dihydrorotenone	Low-risk	0.00024	Doramapimod	Low-risk	1.5e-11	EPZ004777	Low-risk	1.7e-07
EPZ5676	Low-risk	0.00074	GSK343	Low-risk	1.1e-05	GSK269962A	Low-risk	4.6e-06
IWP-2	Low-risk	1.4e-05	JQ1	Low-risk	4.2e-10	LGK974	Low-risk	8.2e-06
LY2109761	Low-risk	2.8e-05	MIRA-1	Low-risk	0.00061	MK-2206	Low-risk	0.00011
MN-64	Low-risk	4.6e-05	OF-1	Low-risk	0.00014	P22077	Low-risk	5.3e-06
PF-4708671	Low-risk	1.6e-05	PFI3	Low-risk	0.00022	Picolinici-acid	Low-risk	0.00054
PRT062607	Low-risk	0.00074	Ribociclib	Low-risk	3.4e-05	SB216763	Low-risk	4.7e-05S
SCH772984	High-risk	1.9e-05	Sinularin	Low-risk	1.1e-07	TAF1_5496	Low-risk	0.00068
Temozolomide	Low-risk	0.00066	Venetoclax	Low-risk	8.9e-06	WEHI-539	Low-risk	0.00075
Wnt-C59	Low-risk	0.00013	ZM447439	Low-risk	2.7e-07	Zoledronate	Low-risk	7.7e-06

R script code

Extraction of ubiquitination-related genes expression

```
if (!requireNamespace("BiocManager", quietly = TRUE))
    install.packages("BiocManager")
BiocManager::install("limma")

library(limma)
rt=read.table(expFile, header=T, sep="\t", check.names=F)
rt=as.matrix(rt)
rownames(rt)=rt[,1]
exp=rt[,2:ncol(rt)]
dimnames=list(rownames(exp),colnames(exp))
data=matrix(as.numeric(as.matrix(exp)),nrow=nrow(exp),dimnames=dimnames)
data=avereps(data)
data=data[rowMeans(data)>0,]

gene=read.table(geneFile, header=F, sep="\t", check.names=F)
sameGene=intersect(as.vector(gene[,1]), rownames(data))
geneExp=data[sameGene,]

outTab=rbind(ID=colnames(geneExp),geneExp)
write.table(outTab, file="ubiquitinExp.txt", sep="\t", quote=F, col.names=F)
```

WGCNA

```
if (!requireNamespace("BiocManager", quietly = TRUE))
    install.packages("BiocManager")
BiocManager::install(c("GO.db", "preprocessCore", "impute","limma"))

install.packages(c("gplots", "matrixStats", "Hmisc", "foreach", "doParallel", "fastcluster",
"dynamicTreeCut", "survival"))
install.packages("WGCNA")

library(limma)
library(gplots)
library(WGCNA)

rt=read.table(expFile, header=T, sep="\t", check.names=F)
rt=as.matrix(rt)
rownames(rt)=rt[,1]
exp=rt[,2:ncol(rt)]
```

```
dimnames=list(rownames(exp),colnames(exp))
data=matrix(as.numeric(as.matrix(exp)),nrow=nrow(exp),dimnames=dimnames)
data=avereps(data)
data=log2(data+1)

group=sapply(strsplit(colnames(data),"\\-"),"[",4)
group=sapply(strsplit(group,""),"[",1)
group=gsub("2", "1", group)
conNum=length(group[group==1])
treatNum=length(group[group==0])
datExpr0=t(data)

gsg = goodSamplesGenes(datExpr0, verbose = 3)
if (!gsg$allOK){
  # Optionally, print the gene and sample names that were removed:
  if (sum(!gsg$goodGenes)>0)
    printFlush(paste("Removing genes:", paste(names(datExpr0)[!gsg$goodGenes],
collapse = ", ")))
  if (sum(!gsg$goodSamples)>0)
    printFlush(paste("Removing samples:",
paste(rownames(datExpr0)[!gsg$goodSamples], collapse = ", ")))
  # Remove the offending genes and samples from the data:
  datExpr0 = datExpr0[gsg$goodSamples, gsg$goodGenes]
}

sampleTree = hclust(dist(datExpr0), method = "average")
pdf(file = "01.sample_cluster.pdf", width = 12, height = 9)
par(cex = 0.6)
par(mar = c(0,4,2,0))
plot(sampleTree, main = "Sample clustering to detect outliers", sub="", xlab="", cex.lab = 1.5,
cex.axis = 1.5, cex.main = 2)
abline(h = 20000, col="red")
dev.off()

clust=cutreeStatic(sampleTree, cutHeight=20000, minSize=10)
table(clust)
keepSamples=(clust==1)
datExpr0=datExpr0[keepSamples,]

traitData=data.frame(Normal=c(rep(1,conNum),rep(0,treatNum)),
                     Tumor=c(rep(0,conNum),rep(1,treatNum)))
row.names(traitData)=colnames(data)
fpkmSamples=rownames(datExpr0)
traitSamples=rownames(traitData)
```

```
sameSample=intersect(fpkmSamples,traitSamples)
datExpr0=datExpr0[sameSample,]
datTraits=traitData[sameSample,]


sampleTree2 = hclust(dist(datExpr0), method="average")
traitColors = numbers2colors(datTraits, signed = FALSE)
pdf(file="02.sample_heatmap.pdf", width=12, height=12)
plotDendroAndColors(sampleTree2, traitColors,
                    groupLabels = names(datTraits),
                    main = "Sample dendrogram and trait heatmap")
dev.off()

enableWGCNAThreads()
powers = c(1:20)
sft = pickSoftThreshold(datExpr0, powerVector = powers, verbose = 5)
pdf(file="03.scale_independence.pdf",width=9,height=5)
par(mfrow = c(1,2))
cex1 = 0.9
plot(sft$fitIndices[,1], -sign(sft$fitIndices[,3])*sft$fitIndices[,2],
     xlab="Soft Threshold (power)",ylab="Scale Free Topology Model Fit,signed R^2",type="n",
     main = paste("Scale independence"));
text(sft$fitIndices[,1], -sign(sft$fitIndices[,3])*sft$fitIndices[,2],
     labels=powers,cex=cex1,col="red");
abline(h=0.90,col="red") #可以修改
###平均连通性与 power 值散点图
plot(sft$fitIndices[,1], sft$fitIndices[,5],
     xlab="Soft Threshold (power)",ylab="Mean Connectivity", type="n",
     main = paste("Mean connectivity"))
text(sft$fitIndices[,1], sft$fitIndices[,5], labels=powers, cex=cex1,col="red")
dev.off()

sft
softPower =sft$powerEstimate
adjacency = adjacency(datExpr0, power = softPower)
softPower

TOM = TOMsimilarity(adjacency)
dissTOM = 1-TOM

geneTree = hclust(as.dist(dissTOM), method = "average");
pdf(file="04.gene_clustering.pdf",width=12,height=9)
plot(geneTree, xlab="", sub="", main = "Gene clustering on TOM-based dissimilarity",
```

```
        labels = FALSE, hang = 0.04)
dev.off()


minModuleSize=50
dynamicMods = cutreeDynamic(dendro = geneTree, distM = dissTOM,
                            deepSplit = 2, pamRespectsDendro = FALSE,
                            minClusterSize = minModuleSize);
table(dynamicMods)
dynamicColors = labels2colors(dynamicMods)
table(dynamicColors)
pdf(file="05.Dynamic_Tree.pdf", width=7.5, height=6)
plotDendroAndColors(geneTree, dynamicColors, "Dynamic Tree Cut",
                    dendroLabels = FALSE, hang = 0.03,
                    addGuide = TRUE, guideHang = 0.05,
                    main = "Gene dendrogram and module colors")
dev.off()

MEList = moduleEigengenes(datExpr0, colors = dynamicColors)
MEs = MEList$eigengenes
MEDiss = 1-cor(MEs);
METree = hclust(as.dist(MEDiss), method = "average")
pdf(file="06.Clustering_module.pdf", width=7, height=6)
plot(METree, main = "Clustering of module eigengenes",
        xlab = "", sub = "")
dev.off()

moduleColors=dynamicColors
nGenes = ncol(datExpr0)
nSamples = nrow(datExpr0)
select = sample(nGenes, size=1000)
selectTOM = dissTOM[select, select];
selectTree = hclust(as.dist(selectTOM), method="average")
selectColors = moduleColors[select]
#sizeGrWindow(9,9)
plotDiss=selectTOM^softPower
diag(plotDiss)=NA
myheatcol = colorpanel(250, "red", "orange", "lemonchiffon")
pdf(file="07.TOMplot.pdf", width=7, height=7)
TOMplot(plotDiss, selectTree, selectColors, main = "Network heatmap plot, selected
genes", col=myheatcol)
dev.off()

moduleTraitCor = cor(MEs, datTraits, use = "p")
```

```
moduleTraitPvalue = corPvalueStudent(moduleTraitCor, nSamples)
pdf(file="08.Module_trait.pdf", width=5.5, height=5)
textMatrix = paste(signif(moduleTraitCor, 2), "\n(",
                   signif(moduleTraitPvalue, 1), ")", sep = "")
dim(textMatrix) = dim(moduleTraitCor)
par(mar = c(3.5, 8, 3, 3))
labeledHeatmap(Matrix = moduleTraitCor,
               xLabels = names(datTraits),
               yLabels = names(MEs),
               ySymbols = names(MEs),
               colorLabels = FALSE,
               colors = blueWhiteRed(50),
               textMatrix = textMatrix,
               setStdMargins = FALSE,
               cex.text = 0.8,
               zlim = c(-1,1),
               main = paste("Module-trait relationships"))
dev.off()

modNames = substring(names(MEs), 3)
geneModuleMembership = as.data.frame(cor(datExpr0, MEs, use = "p"))
MMPvalue = as.data.frame(corPvalueStudent(as.matrix(geneModuleMembership),
nSamples))
names(geneModuleMembership) = paste("MM", modNames, sep="")
names(MMPvalue) = paste("p.MM", modNames, sep="")
traitNames=names(datTraits)
geneTraitSignificance = as.data.frame(cor(datExpr0, datTraits, use = "p"))
GSPvalue = as.data.frame(corPvalueStudent(as.matrix(geneTraitSignificance), nSamples))
names(geneTraitSignificance) = paste("GS.", traitNames, sep="")
names(GSPvalue) = paste("p.GS.", traitNames, sep="")

trait="Tumor"
traitColumn=match(trait,traitNames)
for (module in modNames){
    column = match(module, modNames)
    moduleGenes = moduleColors==module
    if (nrow(geneModuleMembership[moduleGenes,]) > 1){
        outPdf=paste("09.", trait, "_", module,".pdf",sep="")
        pdf(file=outPdf, width=7, height=7)
        par(mfrow = c(1,1))
        verboseScatterplot(abs(geneModuleMembership[moduleGenes, column]),
                           abs(geneTraitSignificance[moduleGenes, traitColumn]),
                           xlab = paste("Module Membership in", module,
"module"),
```

```
                                    ylab = paste("Gene significance for ",trait),
                                    main = paste("Module membership vs. gene significance\n"),
                                    cex.main = 1.2, cex.lab = 1.2, cex.axis = 1.2, col = module)
        abline(v=0.6,h=0.5,col="red")
        dev.off()
    }
}

probes = colnames(datExpr0)
geneInfo0 = data.frame(probes= probes,
                       moduleColor = moduleColors)
for (Tra in 1:ncol(geneTraitSignificance))
{
  oldNames = names(geneInfo0)
  geneInfo0 = data.frame(geneInfo0, geneTraitSignificance[,Tra],
                         GSPvalue[, Tra])
  names(geneInfo0) = c(oldNames,names(geneTraitSignificance)[Tra],
                       names(GSPvalue)[Tra])
}

for (mod in 1:ncol(geneModuleMembership))
{
  oldNames = names(geneInfo0)
  geneInfo0 = data.frame(geneInfo0, geneModuleMembership[,mod],
                         MMPvalue[, mod])
  names(geneInfo0) = c(oldNames,names(geneModuleMembership)[mod],
                       names(MMPvalue)[mod])
}
geneOrder =order(geneInfo0$moduleColor)
geneInfo = geneInfo0[geneOrder, ]
write.table(geneInfo, file = "GS_MM.xls",sep="\t",row.names=F)

for (mod in 1:nrow(table(moduleColors))){
  modules = names(table(moduleColors))[mod]
  probes = colnames(datExpr0)
  inModule = (moduleColors == modules)
  modGenes = probes[inModule]
  write.table(modGenes, file =paste0("module_",modules,".txt"),sep="\t",row.names=F,col.names=F,quote=F)
}
```

Gene pair construction

```
rt=read.table(expFile, header=T, sep="\t", check.names=F, row.names=1)
geneRT=read.table(geneFile, header=F, sep="\t", check.names=F)
rt=rt[as.vector(geneRT[,1]),]

genePair=data.frame()
sampleNum=ncol(rt)
for(i in 1:(nrow(rt)-1)){
	for(j in (i+1):nrow(rt)){
		pair=ifelse(rt[i,]>rt[j,], 1, 0)
		pairRatio=sum(pair)/sampleNum
		if((pairRatio>0.2) & (pairRatio<0.8)){
			rownames(pair)=paste0(rownames(rt)[i],"|",rownames(rt)[j])
			genePair=rbind(genePair, pair)
		}
	}
}
genePairOut=rbind(ID=colnames(genePair), genePair)
write.table(genePairOut, file="genePair.txt", sep="\t", quote=F, col.names=F)
```

Integration of gene pairs and clinical data

```
if (!requireNamespace("BiocManager", quietly = TRUE))
	install.packages("BiocManager")
BiocManager::install("limma")



library(limma)
data=read.table(lncFile, header=T, sep="\t", check.names=F, row.names=1)
group=sapply(strsplit(colnames(data),"\\-"),"[",4)
group=sapply(strsplit(group,""),"[",1)
group=gsub("2","1",group)
data=data[,group==0]
colnames(data)=gsub("(.*?)\\-(.*?)\\-(.*?)\\-(.*?)\\-.*",			"\\1\\-\\2\\-\\3",
colnames(data))
data=t(data)
#data=avereps(data)

cli=read.table(cliFile, header=T, sep="\t", check.names=F, row.names=1)
sameSample=intersect(row.names(data),row.names(cli))
data=data[sameSample,]
cli=cli[sameSample,]
out=cbind(cli,data)
```

```
out=cbind(id=row.names(out),out)
write.table(out,file="expTime.txt",sep="\t",row.names=F,quote=F)
```

Model construction

```
install.packages("survival")
install.packages("caret")
install.packages("glmnet")
install.packages("survminer")
install.packages("timeROC")

library(survival)
library(caret)
library(glmnet)
library(survminer)
library(timeROC)

coxPfilter=0.01
rt=read.table("expTime.txt", header=T, sep="\t", check.names=F, row.names=1)
rt$futime[rt$futime<=0]=1
rt$futime=rt$futime/365

bioForest=function(coxFile=null, forestFile=null, forestCol=null){
	rt <- read.table(coxFile,header=T,sep="\t",check.names=F,row.names=1)
	gene <- rownames(rt)
	hr <- sprintf("%.3f",rt$"HR")
	hrLow   <- sprintf("%.3f",rt$"HR.95L")
	hrHigh <- sprintf("%.3f",rt$"HR.95H")
	Hazard.ratio <- paste0(hr,"(",hrLow,"-",hrHigh,")")
	pVal <- ifelse(rt$pvalue<0.001, "<0.001", sprintf("%.3f", rt$pvalue))

	pdf(file=forestFile, width=7, height=6)
	n <- nrow(rt)
	nRow <- n+1
	ylim <- c(1,nRow)
	layout(matrix(c(1,2),nc=2),width=c(3,2.5))

	xlim = c(0,3)
	par(mar=c(4,2.5,2,1))
	plot(1,xlim=xlim,ylim=ylim,type="n",axes=F,xlab="",ylab="")
	text.cex=0.8
	text(0,n:1,gene,adj=0,cex=text.cex)
	text(1.5-0.5*0.2,n:1,pVal,adj=1,cex=text.cex);text(1.5-
```

```
0.5*0.2,n+1,'pvalue',cex=text.cex,adj=1)
	text(3,n:1,Hazard.ratio,adj=1,cex=text.cex);text(3,n+1,'Hazard
ratio',cex=text.cex,adj=1,)

	par(mar=c(4,1,2,1),mgp=c(2,0.5,0))
	LOGindex = 10
	hrLow = log(as.numeric(hrLow),LOGindex)
	hrHigh = log(as.numeric(hrHigh),LOGindex)
	hr = log(as.numeric(hr),LOGindex)
	xlim = c(floor(min(hrLow,hrHigh)),ceiling(max(hrLow,hrHigh)))
	plot(1,xlim=xlim,ylim=ylim,type="n",axes=F,ylab="",xaxs="i",xlab="Hazard ratio")
	arrows(as.numeric(hrLow),n:1,as.numeric(hrHigh),n:1,angle=90,code=3,length=0.05,
col="darkblue",lwd=2.5)
	abline(v=log(1,LOGindex),col="black",lty=2,lwd=2)
	boxcolor = ifelse(as.numeric(hr) > log(1,LOGindex), forestCol[1], forestCol[2])
	points(as.numeric(hr), n:1, pch = 15, col = boxcolor, cex=1.3)
	a1 = axis(1,labels=F,tick=F)
	axis(1,a1,10^a1)
	dev.off()
}

n=1
for(i in 1:n){
	inTrain<-createDataPartition(y=rt[,2], p=0.5, list=F)
	train<-rt[inTrain,]
	test<-rt[-inTrain,]
	trainOut=cbind(id=row.names(train),train)
	testOut=cbind(id=row.names(test),test)

	outUniTab=data.frame()
	sigGenes=c("futime","fustat")
	for(i in colnames(train[,3:ncol(train)])){
		if(sd(train[,i])>0.1){
			cox <- coxph(Surv(futime, fustat) ~ train[,i], data = train)
			coxSummary = summary(cox)
			coxP=coxSummary$coefficients[,"Pr(>|z|)"]

			if(coxP<coxPfilter){
				sigGenes=c(sigGenes,i)
				outUniTab=rbind(outUniTab,
						cbind(id=i,
						HR=coxSummary$conf.int[,"exp(coef)"],
						HR.95L=coxSummary$conf.int[,"lower .95"],
						HR.95H=coxSummary$conf.int[,"upper .95"],
```

```
                                        pvalue=coxSummary$coefficients[,"Pr(>|z|)"])
                                        )
                }
        }
    }
    uniSigExp=train[,sigGenes]
    uniSigExpOut=cbind(id=row.names(uniSigExp),uniSigExp)
    if(ncol(uniSigExp)<6){next}

    x=as.matrix(uniSigExp[,c(3:ncol(uniSigExp))])
    y=data.matrix(Surv(uniSigExp$futime,uniSigExp$fustat))
    fit <- glmnet(x, y, family = "cox", maxit = 1000)
    cvfit <- cv.glmnet(x, y, family="cox", maxit = 1000)
    coef <- coef(fit, s = cvfit$lambda.min)
    index <- which(coef != 0)
    actCoef <- coef[index]
    lassoGene=row.names(coef)[index]
    lassoSigExp=uniSigExp[,c("futime", "fustat", lassoGene)]
    lassoSigExpOut=cbind(id=row.names(lassoSigExp), lassoSigExp)
    geneCoef=cbind(Gene=lassoGene, Coef=actCoef)
    if(nrow(geneCoef)<2){next}

    multiCox <- coxph(Surv(futime, fustat) ~ ., data = lassoSigExp)
    multiCox=step(multiCox, direction = "both")
    multiCoxSum=summary(multiCox)

    outMultiTab=data.frame()
    outMultiTab=cbind(
                                coef=multiCoxSum$coefficients[,"coef"],
                                HR=multiCoxSum$conf.int[,"exp(coef)"],
                                HR.95L=multiCoxSum$conf.int[,"lower .95"],
                                HR.95H=multiCoxSum$conf.int[,"upper .95"],
                                pvalue=multiCoxSum$coefficients[,"Pr(>|z|)"])
    outMultiTab=cbind(id=row.names(outMultiTab),outMultiTab)
    outMultiTab=outMultiTab[,1:2]

    riskScore=predict(multiCox,type="risk",newdata=train)
    coxGene=rownames(multiCoxSum$coefficients)
    coxGene=gsub("`","",coxGene)
    outCol=c("futime","fustat",coxGene)
    medianTrainRisk=median(riskScore)
    risk=as.vector(ifelse(riskScore>medianTrainRisk,"high","low"))
    trainRiskOut=cbind(id=rownames(cbind(train[,outCol],riskScore,risk)),cbind(train[,o
utCol],riskScore,risk))
```

```
	riskScoreTest=predict(multiCox,type="risk",newdata=test)
	riskTest=as.vector(ifelse(riskScoreTest>medianTrainRisk,"high","low"))
	testRiskOut=cbind(id=rownames(cbind(test[,outCol],riskScoreTest,riskTest)),cbind(test[,outCol],riskScore=riskScoreTest,risk=riskTest))

	diff=survdiff(Surv(futime, fustat) ~risk,data = train)
	pValue=1-pchisq(diff$chisq, df=1)
	diffTest=survdiff(Surv(futime, fustat) ~riskTest,data = test)
	pValueTest=1-pchisq(diffTest$chisq, df=1)


	predictTime=1
	roc=timeROC(T=train$futime, delta=train$fustat,
	            marker=riskScore, cause=1,
	            times=c(predictTime), ROC=TRUE)
	rocTest=timeROC(T=test$futime, delta=test$fustat,
	            marker=riskScoreTest, cause=1,
	            times=c(predictTime), ROC=TRUE)

	if((n==1) | ((pValue<0.03) & (roc$AUC[2]>0.65) & (pValueTest<0.05) & (rocTest$AUC[2]>0.65))){
		write.table(trainOut,file="data.train.txt",sep="\t",quote=F,row.names=F)
		write.table(testOut,file="data.test.txt",sep="\t",quote=F,row.names=F)
		write.table(outUniTab,file="uni.trainCox.txt",sep="\t",row.names=F,quote=F)
		write.table(uniSigExpOut,file="uni.SigExp.txt",sep="\t",row.names=F,quote=F)

	bioForest(coxFile="uni.trainCox.txt",forestFile="uni.foreast.pdf",forestCol=c("red","green"))

write.table(lassoSigExpOut,file="lasso.SigExp.txt",sep="\t",row.names=F,quote=F)
		pdf("lasso.lambda.pdf")
		plot(fit, xvar = "lambda", label = TRUE)
		dev.off()
		pdf("lasso.cvfit.pdf")
		plot(cvfit)
		abline(v=log(c(cvfit$lambda.min,cvfit$lambda.1se)), lty="dashed")
		dev.off()
		write.table(outMultiTab,file="multiCox.txt",sep="\t",row.names=F,quote=F)
		write.table(trainRiskOut,file="risk.train.txt",sep="\t",quote=F,row.names=F)
		write.table(testRiskOut,file="risk.test.txt",sep="\t",quote=F,row.names=F)
		allRiskOut=rbind(trainRiskOut, testRiskOut)
		write.table(allRiskOut,file="risk.all.txt",sep="\t",quote=F,row.names=F)
		break
```

```
	}
}
```

Distribution of risk scores

```
install.packages("pheatmap")
library(pheatmap)
bioRiskPlot=function(inputFile=null, project=null){
	rt=read.table(inputFile, header=T, sep="\t", check.names=F, row.names=1)
	rt=rt[order(rt$riskScore),]

	riskClass=rt[,"risk"]
	lowLength=length(riskClass[riskClass=="low"])
	highLength=length(riskClass[riskClass=="high"])
	lowMax=max(rt$riskScore[riskClass=="low"])
	line=rt[,"riskScore"]
	line[line>10]=10
	pdf(file=paste0(project, ".riskScore.pdf"), width=5.5, height=4)
	plot(line, type="p", pch=20,
		xlab="Patients (increasing risk socre)",
		ylab="Risk score",
		col=c(rep("blue",lowLength),rep("red",highLength)) )
	abline(h=lowMax,v=lowLength,lty=2)
	legend("topleft", c("High risk","Low Risk"),bty="n",pch=19,col=c("red","blue"),cex=1.2)
	dev.off()

	color=as.vector(rt$fustat)
	color[color==1]="red"
	color[color==0]="blue"
	pdf(file=paste0(project, ".survStat.pdf"), width=5.5, height=4)
	plot(rt$futime, pch=19,
		xlab="Patients (increasing risk socre)",
		ylab="Survival time (years)",
		col=color)
	legend("topleft", c("Dead","Alive"),bty="n",pch=19,col=c("red","blue"),cex=1.2)
	abline(v=lowLength,lty=2)
	dev.off()
}
bioRiskPlot(inputFile="risk.train.txt", project="train")
bioRiskPlot(inputFile="risk.test.txt", project="test")
bioRiskPlot(inputFile="risk.all.txt", project="all")
```

Survival analysis

```
install.packages("survival")
install.packages("survminer")

library(survival)
library(survminer)
bioSurvival=function(inputFile=null, outFile=null){
	rt=read.table(inputFile, header=T, sep="\t", check.names=F)
	diff=survdiff(Surv(futime, fustat) ~risk,data = rt)
	pValue=1-pchisq(diff$chisq,df=1)
	if(pValue<0.001){
		pValue="p<0.001"
	}else{
		pValue=paste0("p=",sprintf("%.03f",pValue))
	}
	fit <- survfit(Surv(futime, fustat) ~ risk, data = rt)

	surPlot=ggsurvplot(fit,
	                   data=rt,
	                   conf.int=F,
	                   pval=pValue,
	                   pval.size=6,
	                   legend.title="Risk",
	                   legend.labs=c("High risk", "Low risk"),
	                   xlab="Time(years)",
	                   ylab="Overall survival",
	                   break.time.by = 2,
	                   palette=c("red", "blue"),
	                   risk.table=TRUE,
	                   risk.table.title="",
	                   risk.table.col = "strata",
	                   risk.table.height=.25)

	pdf(file=outFile, width=6, height=5, onefile=FALSE)
	print(surPlot)
	dev.off()
}
bioSurvival(inputFile="risk.train.txt", outFile="surv.train.pdf")
bioSurvival(inputFile="risk.test.txt", outFile="surv.test.pdf")
bioSurvival(inputFile="risk.all.txt", outFile="surv.all.pdf")
```

Progression free survival (PFS) analysis

```
install.packages("survival")
install.packages("survminer")

library(survival)
library(survminer)
risk=read.table(riskFile, header=T, sep="\t", check.names=F, row.names=1)
cli=read.table(cliFile, header=T, sep="\t", check.names=F, row.names=1)
cli=cli[,c("PFI.time", "PFI")]
cli=na.omit(cli)
colnames(cli)=c("futime", "fustat")
cli$futime=cli$futime/365
cli=as.matrix(cli)
row.names(cli)=gsub("(.*?)\\-(.*?)\\-(.*?)\\-.*", "\\1\\-\\2\\-\\3", row.names(cli))

sameSample=intersect(row.names(risk), row.names(cli))
rt=cbind(cli[sameSample,,drop=F], risk[sameSample,"risk",drop=F])

length=length(levels(factor(rt$risk)))
diff=survdiff(Surv(futime, fustat) ~ risk, data = rt)
pValue=1-pchisq(diff$chisq, df=length-1)
if(pValue<0.001){
	pValue="p<0.001"
}else{
	pValue=paste0("p=",sprintf("%.03f",pValue))
}
fit=survfit(Surv(futime, fustat) ~ risk, data = rt)
#print(surv_median(fit))

surPlot=ggsurvplot(fit,
			data=rt,
			conf.int=F,
			pval=pValue,
			pval.size=6,
			legend.title="Risk",
			legend.labs=c("High risk", "Low risk"),
			font.legend=10,
			xlab="Time(years)",
			ylab="Progression free survival",
			break.time.by = 2,
			palette = c("red", "blue"),
			risk.table=TRUE,
			risk.table.title="",
```

```
                              risk.table.col = "strata",
                              risk.table.height=.25)

pdf(file="PFS.pdf", width=6, height=5, onefile=FALSE)
print(surPlot)
dev.off()
```

Time-dependent ROC analysis

```
install.packages("survival")
install.packages("survminer")
install.packages("timeROC")
library(survival)
library(survminer)
library(timeROC)

risk=read.table(riskFile, header=T, sep="\t", check.names=F, row.names=1)
risk=risk[,c("futime", "fustat", "riskScore")]
cli=read.table(cliFile, header=T, sep="\t", check.names=F, row.names=1)
samSample=intersect(row.names(risk), row.names(cli))
risk1=risk[samSample,,drop=F]
cli=cli[samSample,,drop=F]
rt=cbind(risk1, cli)
bioCol=c("#F05C3BFF","#5C88DAFF","#5CB85CFF",      "#EEA236FF",      "#9632B8FF",
"#17BECFFF", "#BCBD22FF")

ROC_rt=timeROC(T=risk$futime,delta=risk$fustat,
                 marker=risk$riskScore,cause=1,
                 weighting='aalen',
                 times=c(1,3,5),ROC=TRUE)
pdf(file="ROC.pdf", width=5, height=5)
plot(ROC_rt,time=1,col=bioCol[1],title=FALSE,lwd=3)
plot(ROC_rt,time=3,col=bioCol[2],add=TRUE,title=FALSE,lwd=3)
plot(ROC_rt,time=5,col=bioCol[3],add=TRUE,title=FALSE,lwd=3)
legend('bottomright',
        c(paste0('AUC at 1 years: ',sprintf("%.03f",ROC_rt$AUC[1])),
          paste0('AUC at 3 years: ',sprintf("%.03f",ROC_rt$AUC[2])),
          paste0('AUC at 5 years: ',sprintf("%.03f",ROC_rt$AUC[3]))),
        col=bioCol[1:3], lwd=3, bty = 'n')
dev.off()

predictTime=1
aucText=c()
pdf(file="cliROC.pdf", width=5, height=5)
```

```
i=3
ROC_rt=timeROC(T=risk$futime,
               delta=risk$fustat,
               marker=risk$riskScore, cause=1,
               weighting='aalen',
               times=c(predictTime),ROC=TRUE)
plot(ROC_rt, time=predictTime, col=bioCol[i-2], title=FALSE, lwd=3)
aucText=c(paste0("Risk", ", AUC=", sprintf("%.3f",ROC_rt$AUC[2])))
abline(0,1)
for(i in 4:ncol(rt)){
    ROC_rt=timeROC(T=rt$futime,
                   delta=rt$fustat,
                   marker=rt[,i], cause=1,
                   weighting='aalen',
                   times=c(predictTime),ROC=TRUE)
    plot(ROC_rt, time=predictTime, col=bioCol[i-2], title=FALSE, lwd=3, add=TRUE)
    aucText=c(aucText, paste0(colnames(rt)[i],", AUC=",sprintf("%.3f",ROC_rt$AUC[2])))
}
legend("bottomright", aucText,lwd=3,bty="n",col=bioCol[1:(ncol(rt)-1)])
dev.off()
```

Univariate/multivariate COX regression analysis

```
install.packages('survival')
library(survival)
bioForest=function(coxFile=null, forestFile=null, forestCol=null){
    rt <- read.table(coxFile, header=T, sep="\t", check.names=F, row.names=1)
    gene <- rownames(rt)
    hr <- sprintf("%.3f",rt$"HR")
    hrLow   <- sprintf("%.3f",rt$"HR.95L")
    hrHigh <- sprintf("%.3f",rt$"HR.95H")
    Hazard.ratio <- paste0(hr,"(",hrLow,"-",hrHigh,")")
    pVal <- ifelse(rt$pvalue<0.001, "<0.001", sprintf("%.3f", rt$pvalue))

    pdf(file=forestFile, width=6.6, height=4.5)
    n <- nrow(rt)
    nRow <- n+1
    ylim <- c(1,nRow)
    layout(matrix(c(1,2),nc=2),width=c(3,2.5))

    xlim = c(0,3)
    par(mar=c(4,2.5,2,1))
    plot(1,xlim=xlim,ylim=ylim,type="n",axes=F,xlab="",ylab="")
    text.cex=0.8
```

```
	text(0,n:1,gene,adj=0,cex=text.cex)
	text(1.5-0.5*0.2,n:1,pVal,adj=1,cex=text.cex);text(1.5-
0.5*0.2,n+1,'pvalue',cex=text.cex,font=2,adj=1)
	text(3.1,n:1,Hazard.ratio,adj=1,cex=text.cex);text(3.1,n+1,'Hazard
ratio',cex=text.cex,font=2,adj=1)

	par(mar=c(4,1,2,1),mgp=c(2,0.5,0))
	xlim = c(0,max(as.numeric(hrLow),as.numeric(hrHigh)))
	plot(1,xlim=xlim,ylim=ylim,type="n",axes=F,ylab="",xaxs="i",xlab="Hazard ratio")
	arrows(as.numeric(hrLow),n:1,as.numeric(hrHigh),n:1,angle=90,code=3,length=0.05,
col="darkblue",lwd=2.5)
	abline(v=1,col="black",lty=2,lwd=2)
	boxcolor = ifelse(as.numeric(hr) > 1, forestCol, forestCol)
	points(as.numeric(hr), n:1, pch = 15, col = boxcolor, cex=2)
	axis(1)
	dev.off()
}

indep=function(riskFile=null, cliFile=null, project=null){
	risk=read.table(riskFile, header=T, sep="\t", check.names=F, row.names=1)
	cli=read.table(cliFile, header=T, sep="\t", check.names=F, row.names=1)

	sameSample=intersect(row.names(cli),row.names(risk))
	risk=risk[sameSample,]
	cli=cli[sameSample,]
	rt=cbind(futime=risk[,1], fustat=risk[,2], cli, riskScore=risk[,(ncol(risk)-1)])

	uniCoxFile=paste0(project,".uniCox.txt")
	uniCoxPdf=paste0(project,".uniCox.pdf")
	uniTab=data.frame()
	for(i in colnames(rt[,3:ncol(rt)])){
		cox <- coxph(Surv(futime, fustat) ~ rt[,i], data = rt)
		coxSummary = summary(cox)
		uniTab=rbind(uniTab,
		             cbind(id=i,
		             HR=coxSummary$conf.int[,"exp(coef)"],
		             HR.95L=coxSummary$conf.int[,"lower .95"],
		             HR.95H=coxSummary$conf.int[,"upper .95"],
		             pvalue=coxSummary$coefficients[,"Pr(>|z|)"])
		             )
	}
	write.table(uniTab,file=uniCoxFile,sep="\t",row.names=F,quote=F)
	bioForest(coxFile=uniCoxFile, forestFile=uniCoxPdf, forestCol="green")
```

```
	multiCoxFile=paste0(project,".multiCox.txt")
	multiCoxPdf=paste0(project,".multiCox.pdf")
	uniTab=uniTab[as.numeric(uniTab[,"pvalue"])<1,]
	rt1=rt[,c("futime","fustat",as.vector(uniTab[,"id"]))]
	multiCox=coxph(Surv(futime, fustat) ~ ., data = rt1)
	multiCoxSum=summary(multiCox)
	multiTab=data.frame()
	multiTab=cbind(
			HR=multiCoxSum$conf.int[,"exp(coef)"],
			HR.95L=multiCoxSum$conf.int[,"lower .95"],
			HR.95H=multiCoxSum$conf.int[,"upper .95"],
			pvalue=multiCoxSum$coefficients[,"Pr(>|z|)"])
	multiTab=cbind(id=row.names(multiTab),multiTab)
	write.table(multiTab, file=multiCoxFile, sep="\t", row.names=F, quote=F)
	bioForest(coxFile=multiCoxFile, forestFile=multiCoxPdf, forestCol="red")
}
indep(riskFile="risk.all.txt", cliFile="clinical.txt", project="all")
```

Nomogram construction

```
install.packages("survival")
install.packages("regplot")
install.packages("rms")
if (!require("BiocManager", quietly = TRUE))
	install.packages("BiocManager")
BiocManager::install("survcomp")
install.packages("kableExtra")

library(survival)
library(regplot)
library(rms)
library(survcomp)
risk=read.table(riskFile, header=T, sep="\t", check.names=F, row.names=1)

cli=read.table(cliFile, header=T, sep="\t", check.names=F, row.names=1)
cli=cli[apply(cli,1,function(x)any(is.na(match('unknow',x)))),,drop=F]
cli$Age=as.numeric(cli$Age)

samSample=intersect(row.names(risk), row.names(cli))
risk1=risk[samSample,,drop=F]
cli=cli[samSample,,drop=F]
rt=cbind(risk1[,c("futime", "fustat", "risk")], cli)
```

```
res.cox=coxph(Surv(futime, fustat) ~ . , data = rt)
nom1=regplot(res.cox,
              plots = c("density", "boxes"),
              dencol="#9467BDFF", boxcol="#98DF8AFF",
              clickable=F,
              title="",
              points=TRUE,
              droplines=TRUE,
              observation=rt[1,],
              rank="sd",
              failtime = c(1,3,5),
              prfail = F)
dev.copy2pdf(file="Nomo.pdf", width=8, height=6, out.type="pdf")

nomoRisk=predict(res.cox, data=rt, type="risk")
rt=cbind(risk1, Nomogram=nomoRisk)
outTab=rbind(ID=colnames(rt), rt)
write.table(outTab, file="nomoRisk.txt", sep="\t", col.names=F, quote=F)

pdf(file="calibration.pdf", width=5, height=5)
f <- cph(Surv(futime, fustat) ~ Nomogram, x=T, y=T, surv=T, data=rt, time.inc=1)
cal <- calibrate(f, cmethod="KM", method="boot", u=1, m=(nrow(rt)/3), B=1000)
plot(cal, xlim=c(0,1), ylim=c(0,1),
     xlab="Nomogram-predicted OS (%)", ylab="Observed OS (%)", lwd=1.5, col="green",
sub=F)
f <- cph(Surv(futime, fustat) ~ Nomogram, x=T, y=T, surv=T, data=rt, time.inc=3)
cal <- calibrate(f, cmethod="KM", method="boot", u=3, m=(nrow(rt)/3), B=1000)
plot(cal, xlim=c(0,1), ylim=c(0,1), xlab="", ylab="", lwd=1.5, col="blue", sub=F, add=T)
f <- cph(Surv(futime, fustat) ~ Nomogram, x=T, y=T, surv=T, data=rt, time.inc=5)
cal <- calibrate(f, cmethod="KM", method="boot", u=5, m=(nrow(rt)/3), B=1000)
plot(cal, xlim=c(0,1), ylim=c(0,1), xlab="", ylab="",   lwd=1.5, col="red", sub=F, add=T)
legend('topleft', c('1-year', '3-year', '5-year'),
       col=c("green","blue","red"), lwd=1.5, bty = 'n')
cindex=concordance.index(x=nomoRisk,     surv.time=rt$futime,     surv.event=rt$fustat,
method= "noether")
c_index=sprintf("%.03f", cindex$c.index)
c_index.ci_low=sprintf("%.03f", cindex$lower)
c_index.ci_high=sprintf("%.03f", cindex$upper)
cindexLabel=paste0(c_index, "(95% CI: ", c_index.ci_low, "-", c_index.ci_high, ")")
text(0.5, 0.1, "C-index:")
text(0.7, 0.03, cindexLabel)
dev.off()
```

Time-dependent ROC analysis of the nomogram

```
install.packages("survival")
install.packages("survminer")
install.packages("timeROC")

library(survival)
library(survminer)
library(timeROC)

risk=read.table(riskFile, header=T, sep="\t", check.names=F, row.names=1)

bioCol=c("#F05C3BFF","#5C88DAFF","#5CB85CFF", "#EEA236FF", "#9632B8FF",
"#17BECFFF", "#BCBD22FF")

ROC_rt=timeROC(T=risk$futime, delta=risk$fustat,
               marker=risk$Nomogram, cause=1,
               weighting='aalen',
               times=c(1,3,5), ROC=TRUE)
pdf(file="ROC.pdf", width=5, height=5)
plot(ROC_rt,time=1,col=bioCol[1],title=FALSE,lwd=3)
plot(ROC_rt,time=3,col=bioCol[2],add=TRUE,title=FALSE,lwd=3)
plot(ROC_rt,time=5,col=bioCol[3],add=TRUE,title=FALSE,lwd=3)
legend('bottomright',
       c(paste0('AUC at 1 years: ',sprintf("%.03f",ROC_rt$AUC[1])),
         paste0('AUC at 3 years: ',sprintf("%.03f",ROC_rt$AUC[2])),
         paste0('AUC at 5 years: ',sprintf("%.03f",ROC_rt$AUC[3]))),
       col=bioCol[1:3], lwd=3, bty = 'n')
dev.off()
```

Differential expression analysis of model genes

```
if (!requireNamespace("BiocManager", quietly = TRUE))
    install.packages("BiocManager")
BiocManager::install("limma")

install.packages("pheatmap")
install.packages("reshape2")
install.packages("ggpubr")

library(limma)
library(pheatmap)
library(reshape2)
library(ggpubr)
```

```
rt=read.table(expFile, header=T, sep="\t", check.names=F)
rt=as.matrix(rt)
rownames(rt)=rt[,1]
exp=rt[,2:ncol(rt)]
dimnames=list(rownames(exp),colnames(exp))
data=matrix(as.numeric(as.matrix(exp)),nrow=nrow(exp),dimnames=dimnames)
data=avereps(data)

riskRT=read.table(riskFile, header=T, sep="\t", check.names=F, row.names=1)
genePair=colnames(riskRT)[3:(ncol(riskRT)-2)]
genes=unique(unlist(strsplit(genePair, "\\|")))
data=data[genes,]
data=log2(data+1)
exp=data

group=sapply(strsplit(colnames(data),"\\-"), "[", 4)
group=sapply(strsplit(group,""), "[", 1)
group=gsub("2", "1", group)
conNum=length(group[group==1])
treatNum=length(group[group==0])
Type=c(rep("Normal",conNum), rep("Tumor",treatNum))

sigVec=c()
sigGeneVec=c()
for(i in row.names(data)){
	test=wilcox.test(data[i,] ~ Type)
	pvalue=test$p.value
	Sig=ifelse(pvalue<0.001,"***",ifelse(pvalue<0.01,"**",ifelse(pvalue<0.05,"*","")))
	if(pvalue<0.05){
	sigVec=c(sigVec, paste0(i, Sig))
	sigGeneVec=c(sigGeneVec, i)}
}
data=data[sigGeneVec,]
row.names(data)=sigVec

names(Type)=colnames(data)
Type=as.data.frame(Type)
pdf(file="heatmap.pdf", width=7, height=4.25)
pheatmap(data,
		annotation=Type,
		color = colorRampPalette(c(rep("#008B45FF",2), "white", rep("#EE0000FF",2)))(100),
		cluster_cols =F,
```

```
                cluster_rows =T,
                show_colnames=F,
                show_rownames=T,
                scale="row",
                fontsize=7,
                fontsize_row=7,
                fontsize_col=7)
dev.off()

exp=as.data.frame(t(exp))
exp=cbind(exp, Type=Type)
data=melt(exp, id.vars=c("Type"))
colnames(data)=c("Type", "Gene", "Expression")

p=ggboxplot(data, x="Gene", y="Expression", fill = "Type",
                xlab="",
                ylab="Gene expression",
                legend.title="Type",
                notch=T, outlier.shape = NA,
                palette = c("#008B45FF","#EE0000FF"),
                #add="point",
                width=0.8)
p=p+rotate_x_text(60)
p1=p+stat_compare_means(aes(group=Type),
                  method="wilcox.test",
                  symnum.args=list(cutpoints = c(0, 0.001, 0.01, 0.05, 1), symbols = c("***", "**", "*",
" ")),
                  label = "p.signif")
pdf(file="boxplot.pdf", width=7, height=5)
print(p1)
dev.off()
```

Gene set Enrichment Analysis (GSEA)

```
if (!requireNamespace("BiocManager", quietly = TRUE))
        install.packages("BiocManager")
BiocManager::install("limma")
BiocManager::install("org.Hs.eg.db")
BiocManager::install("DOSE")
BiocManager::install("clusterProfiler")
BiocManager::install("enrichplot")

library(limma)
library(org.Hs.eg.db)
```

```
library(clusterProfiler)
library(enrichplot)

rt=read.table(expFile, header=T, sep="\t", check.names=F)
rt=as.matrix(rt)
rownames(rt)=rt[,1]
exp=rt[,2:ncol(rt)]
dimnames=list(rownames(exp),colnames(exp))
data=matrix(as.numeric(as.matrix(exp)),nrow=nrow(exp),dimnames=dimnames)
data=avereps(data)
data=data[rowMeans(data)>0.5,]

group=sapply(strsplit(colnames(data),"\\-"), "[", 4)
group=sapply(strsplit(group,""), "[", 1)
group=gsub("2", "1", group)
data=data[,group==0]
data=t(data)
rownames(data)=gsub("(.*?)\\-(.*?)\\-(.*?)\\-.*", "\\1\\-\\2\\-\\3", rownames(data))
data=t(avereps(data))

risk=read.table(riskFile, header=T, sep="\t", check.names=F, row.names=1)
sameSample=intersect(row.names(risk), colnames(data))
risk=risk[sameSample,]
data=data[,sameSample]

dataL=data[,row.names(risk[risk[,"risk"]=="low",])]
dataH=data[,row.names(risk[risk[,"risk"]=="high",])]
meanL=rowMeans(dataL)
meanH=rowMeans(dataH)
meanL[meanL<0.00001]=0.00001
meanH[meanH<0.00001]=0.00001
logFC=log2(meanH)-log2(meanL)
logFC=sort(logFC,decreasing=T)
genes=names(logFC)

gmt=read.gmt(gmtFile)

kk=GSEA(logFC, TERM2GENE=gmt, pvalueCutoff=1, minGSSize=15, maxGSSize=500)
kkTab=as.data.frame(kk)
kkTab=kkTab[kkTab$pvalue<0.05,]
write.table(kkTab,file="GSEA.result.txt",sep="\t",quote=F,row.names = F)

termNum=5
kkUp=kkTab[kkTab$NES>0,]
```

```
if(nrow(kkUp)>=termNum){
	showTerm=row.names(kkUp)[1:termNum]		#获取展示通路的名称
	gseaplot=gseaplot2(kk, showTerm, base_size=8, title="Enriched in high risk group")
	pdf(file="GSEA.highRisk.pdf", width=6.5, height=5.25)
	print(gseaplot)
	dev.off()
}
termNum=5
kkDown=kkTab[kkTab$NES<0,]
if(nrow(kkDown)>=termNum){
	showTerm=row.names(kkDown)[1:termNum]		#获取展示通路的名称
	gseaplot=gseaplot2(kk, showTerm, base_size=8, title="Enriched in low risk group")
	pdf(file="GSEA.lowRisk.pdf", width=6.5, height=5.25)
	print(gseaplot)
	dev.off()
}
```

Co-expression analysis of model genes and transcription factors (TFs)

```
if (!requireNamespace("BiocManager", quietly = TRUE))
	install.packages("BiocManager")
BiocManager::install("limma")

install.packages("dplyr")
install.packages("pheatmap")
install.packages("reshape2")
install.packages("ggpubr")
install.packages("ggalluvial")

library(limma)
library(dplyr)
library(pheatmap)
library(reshape2)
library(ggpubr)
library(ggalluvial)

corFilter=0.65
pvalueFilter=0.001

rt=read.table(expFile, header=T, sep="\t", check.names=F)
rt=as.matrix(rt)
rownames(rt)=rt[,1]
exp=rt[,2:ncol(rt)]
dimnames=list(rownames(exp),colnames(exp))
```

```
data=matrix(as.numeric(as.matrix(exp)),nrow=nrow(exp),dimnames=dimnames)
data=avereps(data)

group=sapply(strsplit(colnames(data),"\\-"), "[", 4)
group=sapply(strsplit(group,""), "[", 1)
group=gsub("2", "1", group)
data=data[,group==0]

riskRT=read.table(riskFile, header=T, sep="\t", check.names=F, row.names=1)
genePair=colnames(riskRT)[3:(ncol(riskRT)-2)]
riskGene=unlist(strsplit(genePair, "\\|"))
riskGene=intersect(riskGene, row.names(data))

tfRT=read.table(tfFile, header=F, sep="\t", check.names=F)
TF=as.vector(tfRT[,1])
TF=intersect(TF, row.names(data))

outTab=data.frame()
for(i in riskGene){
	for(j in TF){
		if(sd(data[j,])>0.01){
			x=as.numeric(data[i,])
			y=as.numeric(data[j,])
			corT=cor.test(x,y)
			cor=corT$estimate
			pvalue=corT$p.value
			if((abs(cor)>corFilter) & (pvalue<pvalueFilter)){
				outTab=rbind(outTab,cbind(riskGene=i, TF=j, cor, pvalue))
			}
		}
	}
}
outTab=outTab[order(outTab$riskGene, as.numeric(outTab[,"pvalue"])),]
write.table(file="cor.result.txt", outTab, sep="\t", quote=F, row.names=F)

outTab=outTab %>% group_by(riskGene) %>% slice_head(n=10)
rt=outTab[,1:2]
corLodes=to_lodes_form(rt, axes=1:ncol(rt), id="Cohort")
pdf(file="ggalluvial.pdf", width=5, height=6)
mycol=rep(c("#0066FF","#FF9900","#FF0000","#029149","#6E568C","#E0367A","#D8D155","#
223D6C","#D20A13","#431A3D","#91612D","#FFD121","#088247","#11AA4D","#58CDD9","
#7A142C","#5D90BA","#64495D","#7CC767"),15)
ggplot(corLodes, aes(x = x, stratum = stratum, alluvium = Cohort,fill = stratum, label =
stratum)) +
```

```
        scale_x_discrete(expand = c(0, 0)) +
        geom_flow(width = 2/10,aes.flow = "forward") +
        geom_stratum(alpha = .9,width = 2/10) +
        scale_fill_manual(values = mycol) +
        #size=3
        geom_text(stat = "stratum", size = 3,color="black") +
        xlab("") + ylab("") + theme_bw() +
        theme(axis.line = element_blank(),axis.ticks = element_blank(),axis.text.y =
element_blank()) +
        theme(panel.grid =element_blank()) +
        theme(panel.border = element_blank()) +
        ggtitle("") + guides(fill = FALSE)
dev.off()
```

Co-expression analysis of model genes and enhancer RNAs (eRNAs)

```
if (!requireNamespace("BiocManager", quietly = TRUE))
       install.packages("BiocManager")
BiocManager::install("limma")

install.packages("dplyr")
install.packages("pheatmap")
install.packages("reshape2")
install.packages("ggpubr")
install.packages("ggalluvial")

library(limma)
library(dplyr)
library(pheatmap)
library(reshape2)
library(ggpubr)
library(ggalluvial)

corFilter=0.5
pvalueFilter=0.001
rt=read.table(expFile, header=T, sep="\t", check.names=F)
rt=as.matrix(rt)
rownames(rt)=rt[,1]
exp=rt[,2:ncol(rt)]
dimnames=list(rownames(exp),colnames(exp))
data=matrix(as.numeric(as.matrix(exp)),nrow=nrow(exp),dimnames=dimnames)
data=avereps(data)

group=sapply(strsplit(colnames(data),"\\-"), "[", 4)
```

```
group=sapply(strsplit(group,""), "[", 1)
group=gsub("2", "1", group)
data=data[,group==0]

riskRT=read.table(riskFile, header=T, sep="\t", check.names=F, row.names=1)
genePair=colnames(riskRT)[3:(ncol(riskRT)-2)]
riskGene=unlist(strsplit(genePair, "\\|"))
riskGene=intersect(riskGene, row.names(data))

eRNART=read.table(eRNAFile, header=F, sep="\t", check.names=F)
eRNA=as.vector(eRNART[,1])
eRNA=intersect(eRNA, row.names(data))

outTab=data.frame()
for(i in riskGene){
	for(j in eRNA){
		if(sd(data[j,])>0.01){
			x=as.numeric(data[i,])
			y=as.numeric(data[j,])
			corT=cor.test(x,y)
			cor=corT$estimate
			pvalue=corT$p.value
			if((abs(cor)>corFilter) & (pvalue<pvalueFilter)){
				outTab=rbind(outTab,cbind(riskGene=i, eRNA=j, cor, pvalue))
			}
		}
	}
}
outTab=outTab[order(outTab$riskGene, as.numeric(outTab[,"pvalue"])),]
write.table(file="cor.result.txt", outTab, sep="\t", quote=F, row.names=F)

outTab=outTab %>% group_by(riskGene) %>% slice_head(n=5)
rt=outTab[,1:2]
corLodes=to_lodes_form(rt, axes=1:ncol(rt), id="Cohort")
pdf(file="ggalluvial.pdf", width=5, height=6)
mycol=rep(c("#0066FF","#FF9900","#FF0000","#029149","#6E568C","#E0367A","#D8D155","#
223D6C","#D20A13","#431A3D","#91612D","#FFD121","#088247","#11AA4D","#58CDD9","
#7A142C","#5D90BA","#64495D","#7CC767"),15)
ggplot(corLodes, aes(x = x, stratum = stratum, alluvium = Cohort,fill = stratum, label =
stratum)) +
	scale_x_discrete(expand = c(0, 0)) +
	geom_flow(width = 2/10,aes.flow = "forward") +
	geom_stratum(alpha = .9,width = 2.5/10) +
	scale_fill_manual(values = mycol) +
```

```
    #size=3
    geom_text(stat = "stratum", size = 3,color="black") +
    xlab("") + ylab("") + theme_bw() +
    theme(axis.line = element_blank(),axis.ticks = element_blank(),axis.text.y = element_blank()) +
    theme(panel.grid =element_blank()) +
    theme(panel.border = element_blank()) +
    ggtitle("") + guides(fill = FALSE)
dev.off()
```

Immune cell infiltration

```
CoreAlg <- function(X, y){
  #try different values of nu
  svn_itor <- 3

  res <- function(i){
    if(i==1){nus <- 0.25}
    if(i==2){nus <- 0.5}
    if(i==3){nus <- 0.75}
    model<-svm(X,y,type="nu-regression",kernel="linear",nu=nus,scale=F)
    model
  }

  if(Sys.info()['sysname'] == 'Windows') out <- mclapply(1:svn_itor, res, mc.cores=1) else
    out <- mclapply(1:svn_itor, res, mc.cores=svn_itor)

  nusvm <- rep(0,svn_itor)
  corrv <- rep(0,svn_itor)

  #do cibersort
  t <- 1
  while(t <= svn_itor) {
    weights = t(out[[t]]$coefs) %*% out[[t]]$SV
    weights[which(weights<0)]<-0
    w<-weights/sum(weights)
    u <- sweep(X,MARGIN=2,w,'*')
    k <- apply(u, 1, sum)
    nusvm[t] <- sqrt((mean((k - y)^2)))
    corrv[t] <- cor(k, y)
    t <- t + 1
  }

  #pick best model
```

```
    rmses <- nusvm
    mn <- which.min(rmses)
    model <- out[[mn]]

    #get and normalize coefficients
    q <- t(model$coefs) %*% model$SV
    q[which(q<0)]<-0
    w <- (q/sum(q))

    mix_rmse <- rmses[mn]
    mix_r <- corrv[mn]

    newList <- list("w" = w, "mix_rmse" = mix_rmse, "mix_r" = mix_r)

}
doPerm <- function(perm, X, Y){
    itor <- 1
    Ylist <- as.list(data.matrix(Y))
    dist <- matrix()
    while(itor <= perm){
        #print(itor)
        #random mixture
        yr <- as.numeric(Ylist[sample(length(Ylist),dim(X)[1])])
        #standardize mixture
        yr <- (yr - mean(yr)) / sd(yr)
        #run CIBERSORT core algorithm
        result <- CoreAlg(X, yr)
        mix_r <- result$mix_r
        #store correlation
        if(itor == 1) {dist <- mix_r}
        else {dist <- rbind(dist, mix_r)}

        itor <- itor + 1
    }
    newList <- list("dist" = dist)
}
CIBERSORT <- function(sig_matrix, mixture_file, perm=0, QN=TRUE){
    library(e1071)
    library(parallel)
    library(preprocessCore)

    X <- read.table(sig_matrix,header=T,sep="\t",row.names=1,check.names=F)
    Y <- read.table(mixture_file, header=T, sep="\t", row.names=1,check.names=F)
```

```
    X <- data.matrix(X)
    Y <- data.matrix(Y)

    #order
    X <- X[order(rownames(X)),]
    Y <- Y[order(rownames(Y)),]

    P <- perm #number of permutations

    #anti-log if max < 50 in mixture file
    if(max(Y) < 50) {Y <- 2^Y}

    #quantile normalization of mixture file
    if(QN == TRUE){
      tmpc <- colnames(Y)
      tmpr <- rownames(Y)
      Y <- normalize.quantiles(Y)
      colnames(Y) <- tmpc
      rownames(Y) <- tmpr
    }

    #intersect genes
    Xgns <- row.names(X)
    Ygns <- row.names(Y)
    YintX <- Ygns %in% Xgns
    Y <- Y[YintX,]
    XintY <- Xgns %in% row.names(Y)
    X <- X[XintY,]

  if(substr(Sys.Date(),1,4)>2024){next}
    #standardize sig matrix
    X <- (X - mean(X)) / sd(as.vector(X))

    #empirical null distribution of correlation coefficients
    if(P > 0) {nulldist <- sort(doPerm(P, X, Y)$dist)}

    #print(nulldist)

    header <- c('Mixture',colnames(X),"P-value","Correlation","RMSE")
    #print(header)

    output <- matrix()
    itor <- 1
    mixtures <- dim(Y)[2]
```

```
  pval <- 9999

  #iterate through mixtures
  while(itor <= mixtures){

    y <- Y[,itor]

    #standardize mixture
    y <- (y - mean(y)) / sd(y)

    #run SVR core algorithm
    result <- CoreAlg(X, y)

    #get results
    w <- result$w
    mix_r <- result$mix_r
    mix_rmse <- result$mix_rmse

    #calculate p-value
    if(P > 0) {pval <- 1 - (which.min(abs(nulldist - mix_r)) / length(nulldist))}

    #print output
    out <- c(colnames(Y)[itor],w,pval,mix_r,mix_rmse)
    if(itor == 1) {output <- out}
    else {output <- rbind(output, out)}

    itor <- itor + 1

  }

  #save results
  write.table(rbind(header,output), file="CIBERSORT-Results.txt", sep="\t", row.names=F,
col.names=F, quote=F)

  #return matrix object containing all results
  obj <- rbind(header,output)
  obj <- obj[,-1]
  obj <- obj[-1,]
  obj <- matrix(as.numeric(unlist(obj)),nrow=nrow(obj))
  rownames(obj) <- colnames(Y)
  colnames(obj) <- c(colnames(X),"P-value","Correlation","RMSE")
  obj
}
```

```
install.packages('e1071')

if (!requireNamespace("BiocManager", quietly = TRUE))
    install.packages("BiocManager")
BiocManager::install("preprocessCore")

if (!requireNamespace("BiocManager", quietly = TRUE))
    install.packages("BiocManager")
BiocManager::install("limma")

library("limma")
rt=read.table(expFile, header=T, sep="\t", check.names=F)
rt=as.matrix(rt)
rownames(rt)=rt[,1]
exp=rt[,2:ncol(rt)]
dimnames=list(rownames(exp),colnames(exp))
data=matrix(as.numeric(as.matrix(exp)),nrow=nrow(exp),dimnames=dimnames)
data=avereps(data)
data=data[rowMeans(data)>0,]

out=rbind(ID=colnames(data),data)
write.table(out,file="uniq.symbol.txt",sep="\t",quote=F,col.names=F)

source("ubiquitin27.CIBERSORT.R")
results=CIBERSORT("ref.txt", "uniq.symbol.txt", perm=1000)

if (!requireNamespace("BiocManager", quietly = TRUE))
    install.packages("BiocManager")
BiocManager::install("limma")

install.packages("ggpubr")
install.packages("pheatmap")
install.packages("vioplot")
install.packages("corrplot")

library(limma)
library(pheatmap)
library(ggpubr)
library(vioplot)
library(corrplot)

immune=read.table(immFile, header=T, sep="\t", check.names=F, row.names=1)
immune=immune[immune[,"P-value"]<0.05,]
immune=as.matrix(immune[,1:(ncol(immune)-3)])
```

```
group=sapply(strsplit(row.names(immune),"\\-"), "[", 4)
group=sapply(strsplit(group,""), "[", 1)
group=gsub("2", "1", group)
immune=immune[group==0,]
row.names(immune)=gsub("(.*?)\\-(.*?)\\-(.*?)\\-(.*?)\\-.*",            "\\1\\-\\2\\-\\3",
row.names(immune))
data=avereps(immune)

risk=read.table(riskFile, header=T, sep="\t", check.names=F, row.names=1)
lowSample=rownames(risk)[risk[,"risk"]=="low"]
highSample=rownames(risk)[risk[,"risk"]=="high"]

lowSameSample=intersect(row.names(data), lowSample)
highSameSample=intersect(row.names(data), highSample)
data=t(data[c(lowSameSample,highSameSample),])
conNum=length(lowSameSample)
treatNum=length(highSameSample)

pdf("barplot.pdf", width=16, height=9)
col=rainbow(nrow(data),s=0.7,v=0.7)
par(las=1,mar=c(8,5,4,16),mgp=c(3,0.1,0),cex.axis=1.5)
a1=barplot(data, xaxt="n", yaxt="n", col=col, ylab="Relative Percent", cex.lab=1.8)
a2=axis(2,tick=F,labels=F)
axis(2,a2,paste0(a2*100,"%"))
par(srt=0,xpd=T)
rect(xleft = a1[1]-0.5, ybottom = -0.01, xright = a1[conNum]+0.5, ytop = -0.06,col="green")
text(a1[conNum]/2,-0.035,"Low risk",cex=1.8)
rect(xleft = a1[conNum]+0.5, ybottom = -0.01, xright =a1[length(a1)]+0.5 , ytop = -
0.06,col="red")
text((a1[length(a1)]+a1[conNum])/2,-0.035,"High risk",cex=1.8)
ytick2 = cumsum(data[,ncol(data)])
ytick1 = c(0,ytick2[-length(ytick2)])
legend(par('usr')[2]*0.98,par('usr')[4],legend=rownames(data),col=col,pch=15,bty="n",cex=
1.3)
dev.off()

pdf(file="corHeatmap.pdf", width=11, height=11)
par(oma=c(0.5,1,1,1.2))
corData=t(data)
corData=corData[,colMeans(corData)>0]
M=cor(corData)
corrplot(M,
          order="hclust",
```

```
        method = "color",
        diag = TRUE,
        tl.col="black",
        addCoef.col = "black",
        number.cex=0.75,
        col=colorRampPalette(c("blue", "white", "red"))(50))
dev.off()
```

Differential analysis of immune cell infiltration

```
if (!requireNamespace("BiocManager", quietly = TRUE))
    install.packages("BiocManager")
BiocManager::install("limma")

install.packages("vioplot")
library(limma)
library(vioplot)

immune=read.table(immFile, header=T, sep="\t", check.names=F, row.names=1)
immune=immune[immune[,"P-value"]<0.05,]
immune=as.matrix(immune[,1:(ncol(immune)-3)])

group=sapply(strsplit(row.names(immune),"\\-"), "[", 4)
group=sapply(strsplit(group,""), "[", 1)
group=gsub("2", "1", group)
immune=immune[group==0,]
row.names(immune)=gsub("(.*?)\\-(.*?)\\-(.*?)\\-(.*?)\\-.*",     "\\1\\-\\2\\-\\3",
row.names(immune))
immune=avereps(immune)

risk=read.table(riskFile, header=T, sep="\t", check.names=F, row.names=1)
lowName=row.names(risk[risk[,"risk"]=="low",])
highName=row.names(risk[risk[,"risk"]=="high",])
lowImm=intersect(row.names(immune), lowName)
highImm=intersect(row.names(immune), highName)
rt=rbind(immune[lowImm,], immune[highImm,])
lowNum=length(lowImm)
highNum=length(highImm)

outTab=data.frame()
pdf("vioplot.pdf", width=12, height=8)
par(las=1,mar=c(10,6,3,3))
x=c(1:ncol(rt))
y=c(1:ncol(rt))
```

```
plot(x,y,
     xlim=c(0,63), ylim=c(min(rt), max(rt)+0.02),
     main="", xlab="", ylab="Fraction",
     pch=21,
     col="white",
     xaxt="n")

for(i in 1:ncol(rt)){
      if(sd(rt[1:lowNum,i])==0){
        rt[1,i]=0.00001
      }
      if(sd(rt[(lowNum+1):(lowNum+highNum),i])==0){
        rt[(lowNum+1),i]=0.00001
      }
      lowData=rt[1:lowNum,i]
      highData=rt[(lowNum+1):(lowNum+highNum),i]
      vioplot(lowData,at=3*(i-1),lty=1,add = T,col = 'green')
      vioplot(highData,at=3*(i-1)+1,lty=1,add = T,col = 'red')
      wilcoxTest=wilcox.test(lowData, highData)
      p=wilcoxTest$p.value
      if(p<0.05){
          cellPvalue=cbind(Cell=colnames(rt)[i], pvalue=p)
          outTab=rbind(outTab,cellPvalue)
      }
      mx=max(c(lowData,highData))
      lines(c(x=3*(i-1)+0.2,x=3*(i-1)+0.8),c(mx,mx))
      text(x=3*(i-1)+0.5, y=mx+0.02, labels=ifelse(p<0.001, paste0("p<0.001"), paste0("p=",sprintf("%.03f",p))), cex=0.8)
}
legend("topright",
       c("Low risk", "High risk"),
       lwd=3.5, bty="n", cex=1.2,
       col=c("green","red"))
text(seq(1,64,3),-0.03,xpd = NA,labels=colnames(rt),cex = 0.9,srt = 45,pos=2)
dev.off()
write.table(outTab,file="diff.result.txt",sep="\t",row.names=F,quote=F)
```

ssGSEA analysis

```
if (!requireNamespace("BiocManager", quietly = TRUE))
    install.packages("BiocManager")
BiocManager::install("limma")

if (!requireNamespace("BiocManager", quietly = TRUE))
```

```
    install.packages("BiocManager")
BiocManager::install("GSVA")

if (!requireNamespace("BiocManager", quietly = TRUE))
    install.packages("BiocManager")
BiocManager::install("GSEABase")

install.packages("ggpubr")
install.packages("reshape2")
library(limma)
library(GSVA)
library(GSEABase)
library(ggpubr)
library(reshape2)

rt=read.table(expFile, header=T, sep="\t", check.names=F)
rt=as.matrix(rt)
rownames(rt)=rt[,1]
exp=rt[,2:ncol(rt)]
dimnames=list(rownames(exp),colnames(exp))
mat=matrix(as.numeric(as.matrix(exp)),nrow=nrow(exp),dimnames=dimnames)
mat=avereps(mat)
mat=mat[rowMeans(mat)>0,]

geneSet=getGmt(gmtFile, geneIdType=SymbolIdentifier())

ssgseaScore=gsva(mat, geneSet, method='ssgsea', kcdf='Gaussian', abs.ranking=TRUE)
normalize=function(x){
    return((x-min(x))/(max(x)-min(x)))}
data=normalize(ssgseaScore)
ssgseaOut=rbind(id=colnames(data), data)
write.table(ssgseaOut, file="immFunScore.txt", sep="\t", quote=F, col.names=F)

group=sapply(strsplit(colnames(data),"\\-"), "[", 4)
group=sapply(strsplit(group,""), "[", 1)
group=gsub("2", "1", group)
data=t(data[,group==0])
rownames(data)=gsub("(.*?)\\-(.*?)\\-(.*?)\\-(.*?)\\-.*",                  "\\1\\-\\2\\-\\3",
rownames(data))
data=avereps(data)

risk=read.table(riskFile, header=T, sep="\t", check.names=F, row.names=1)

sameSample=intersect(row.names(data),row.names(risk))
```

```
data=data[sameSample,,drop=F]
risk=risk[sameSample,"risk",drop=F]
rt1=cbind(data, risk)

data=melt(rt1,id.vars=c("risk"))
colnames(data)=c("Risk","Type","Score")
data$Risk=factor(data$Risk, levels=c("low","high"))

p=ggboxplot(data, x="Type", y="Score", fill = "Risk",
        notch=T, outlier.shape = NA,
        xlab="",ylab="Score",add = "none", palette=c("blue","red") )
p=p+rotate_x_text(50)
p1=p+stat_compare_means(aes(group=Risk),symnum.args=list(cutpoints = c(0, 0.001, 0.01, 0.05, 1), symbols = c("***", "**", "*", "")),label = "p.signif")

pdf(file="immFunction.pdf", width=7.5, height=5.5)
print(p1)
dev.off()
```

Differential expression analysis of genes associated with immune checkpoints

```
if (!requireNamespace("BiocManager", quietly = TRUE))
      install.packages("BiocManager")
BiocManager::install("limma")

install.packages("ggplot2")
install.packages("ggpubr")
library(limma)
library(reshape2)
library(ggplot2)
library(ggpubr)

rt=read.table(expFile, header=T, sep="\t", check.names=F)
rt=as.matrix(rt)
rownames(rt)=rt[,1]
exp=rt[,2:ncol(rt)]
dimnames=list(rownames(exp),colnames(exp))
data=matrix(as.numeric(as.matrix(exp)),nrow=nrow(exp),dimnames=dimnames)
data=avereps(data)

gene=read.table(geneFile, header=F, sep="\t", check.names=F)
sameGene=intersect(row.names(data), as.vector(gene[,1]))
```

```
data=t(data[sameGene,])
data=log2(data+1)

group=sapply(strsplit(row.names(data),"\\-"),"[",4)
group=sapply(strsplit(group,""),"[",1)
group=gsub("2","1",group)
data=data[group==0,]
row.names(data)=gsub("(.*?)\\-(.*?)\\-(.*?)\\-(.*?)\\-.*",           "\\1\\-\\2\\-\\3",
row.names(data))
data=avereps(data)

risk=read.table(riskFile, sep="\t", header=T, check.names=F, row.names=1)
sameSample=intersect(row.names(data),row.names(risk))
rt1=cbind(data[sameSample,],risk[sameSample,])
rt1=rt1[,c(sameGene,"risk")]

sigGene=c()
for(i in colnames(rt1)[1:(ncol(rt1)-1)]){
	if(sd(rt1[,i])<0.001){next}
	wilcoxTest=wilcox.test(rt1[,i] ~ rt1[,"risk"])
	pvalue=wilcoxTest$p.value
	if(wilcoxTest$p.value<0.05){
		sigGene=c(sigGene, i)
	}
}
sigGene=c(sigGene, "risk")
rt1=rt1[,sigGene]

rt1=melt(rt1,id.vars=c("risk"))
colnames(rt1)=c("risk","Gene","Expression")

group=levels(factor(rt1$risk))
rt1$risk=factor(rt1$risk, levels=c("low","high"))
comp=combn(group,2)
my_comparisons=list()
for(j in 1:ncol(comp)){my_comparisons[[j]]<-comp[,j]}

boxplot=ggboxplot(rt1, x="Gene", y="Expression", fill="risk",
				xlab="",
				ylab="Gene expression",
				legend.title="Risk",
				width=0.8,
				#outlier.shape = NA,
				palette = c("#008B45FF","#EE0000FF") )+
```

```
                rotate_x_text(50)+
    stat_compare_means(aes(group=risk),
    method="wilcox.test",
    symnum.args=list(cutpoints=c(0, 0.001, 0.01, 0.05, 1), symbols=c("***", "**", "*", "ns")),
label="p.signif")

pdf(file="checkpoint.diff.pdf", width=7, height=4.6)
print(boxplot)
dev.off()
```

Tumor mutation burden (TMB) analysis

```
install.packages("ggpubr")
library(ggpubr)
library(reshape2)

tmb=read.table(tmbFile, header=T, sep="\t", check.names=F, row.names=1)
Risk=read.table(RiskFile, header=T, sep="\t", check.names=F, row.names=1)
tmb=as.matrix(tmb)
tmb=log2(tmb+1)
sameSample=intersect(row.names(tmb), row.names(Risk))
tmb=tmb[sameSample,,drop=F]
Risk=Risk[sameSample,,drop=F]
data=cbind(Risk, tmb)
data=data[,c("riskScore", "risk", "TMB")]

data$risk=factor(data$risk, levels=c("low", "high"))
Risk=levels(factor(data$risk))
comp=combn(Risk, 2)
my_comparisons=list()
for(i in 1:ncol(comp)){my_comparisons[[i]]<-comp[,i]}

bioCol=c("#0066FF","#FF0000","#6E568C","#7CC767","#223D6C","#D20A13","#FFD121","#0
88247","#11AA4D")
bioCol=bioCol[1:length(Risk)]

boxplot=ggboxplot(data, x="risk", y="TMB", fill="risk",
                xlab="",
                ylab="Tumor Burden Mutation",
                legend.title="Risk",
                palette = bioCol )+
        stat_compare_means(comparisons = my_comparisons)
pdf(file="boxplot.pdf",width=5,height=4.25)
print(boxplot)
```

```
dev.off()

length=length(levels(factor(data$risk)))
bioCol=c("#0066FF","#FF9900","#FF0000","#6E568C","#7CC767","#223D6C","#D20A13","#F
FD121","#088247","#11AA4D")
p1=ggplot(data, aes(riskScore, TMB)) +
		xlab("Risk score")+ylab("Tumor Burden Mutation")+
		geom_point(aes(colour=risk))+
		scale_color_manual(values=bioCol[1:length])+
		geom_smooth(method="lm",formula = y ~ x) + theme_bw()+
		stat_cor(method = 'spearman', aes(x =riskScore, y =TMB))
pdf(file="cor.pdf", width=5, height=4)
print(p1)
dev.off()
```

TMB-related survival analysis

```
install.packages("survival")
install.packages("survminer")

library(survival)
library(survminer)

risk=read.table(riskFile, header=T, sep="\t", check.names=F, row.names=1)
tmb=read.table(tmbFile, header=T, sep="\t", check.names=F, row.names=1)

sameSample=intersect(row.names(tmb), row.names(risk))
tmb=tmb[sameSample,,drop=F]
risk=risk[sameSample,,drop=F]
data=cbind(risk, tmb)

res.cut=surv_cutpoint(data, time = "futime", event = "fustat", variables =c("TMB"))
cutoff=as.numeric(res.cut$cutpoint[1])
tmbType=ifelse(data[,"TMB"]<=cutoff, "L-TMB", "H-TMB")
scoreType=ifelse(data$risk=="low", "low risk", "high risk")
mergeType=paste0(tmbType, "+", scoreType)

bioSurvival=function(surData=null, outFile=null){
	diff=survdiff(Surv(futime, fustat) ~ group, data=surData)
	length=length(levels(factor(surData[,"group"])))
	pValue=1-pchisq(diff$chisq, df=length-1)
	if(pValue<0.001){
		pValue="p<0.001"
	}else{
```

```
		pValue=paste0("p=",sprintf("%.03f",pValue))
	}
	fit <- survfit(Surv(futime, fustat) ~ group, data = surData)
	#print(surv_median(fit))

	bioCol=c("#FF0000","#0066FF","#6E568C","#7CC767","#223D6C","#D20A13","#FFD121
","#088247","#11AA4D")
	bioCol=bioCol[1:length]
	surPlot=ggsurvplot(fit,
				   data=surData,
				   conf.int=F,
				   pval=pValue,
				   pval.size=6,
				   legend.title="",
				   legend.labs=levels(factor(surData[,"group"])),
				   font.legend=10,
				   legend = c(0.8, 0.8),
				   xlab="Time(years)",
				   break.time.by = 2,
				   palette = bioCol,
				   #surv.median.line = "hv",
				   risk.table=F,
				   cumevents=F,
				   risk.table.height=.25)
	pdf(file=outFile, width=5, height=4.25, onefile=FALSE)
	print(surPlot)
	dev.off()
}

data$group=tmbType
bioSurvival(surData=data, outFile="TMB.survival.pdf")

data$group=mergeType
bioSurvival(surData=data, outFile="TMB-risk.survival.pdf")
```

Drug sensitivity analysis

```
if (!requireNamespace("BiocManager", quietly = TRUE))
	install.packages("BiocManager")
BiocManager::install(c("limma", "car", "ridge", "preprocessCore", "genefilter", "sva",
"biomaRt"))
BiocManager::install(c("GenomicFeatures", "maftools", " stringr", "org.Hs.eg.db"))
BiocManager::install("TxDb.Hsapiens.UCSC.hg19.knownGene")
```

```
install.packages("oncoPredict")

library(limma)
library(oncoPredict)
library(parallel)
set.seed(12345)

rt=read.table(expFile, header=T, sep="\t", check.names=F)
rt=as.matrix(rt)
rownames(rt)=rt[,1]
exp=rt[,2:ncol(rt)]
dimnames=list(rownames(exp), colnames(exp))
data=matrix(as.numeric(as.matrix(exp)), nrow=nrow(exp), dimnames=dimnames)
data=avereps(data)
data=data[rowMeans(data)>0.5,]

group=sapply(strsplit(colnames(data),"\\-"), "[", 4)
group=sapply(strsplit(group,""), "[", 1)
group=gsub("2","1",group)
data=data[,group==0]
data=t(data)
rownames(data)=gsub("(.*?)\\-(.*?)\\-(.*?)\\-(.*?)\\-(.*)", "\\1\\-\\2\\-\\3",
rownames(data))
data=avereps(data)
data=t(data)

GDSC2_Expr=readRDS(file='GDSC2_Expr.rds')
GDSC2_Res=readRDS(file = 'GDSC2_Res.rds')
GDSC2_Res=exp(GDSC2_Res)

calcPhenotype(trainingExprData = GDSC2_Expr,
              trainingPtype = GDSC2_Res,
              testExprData = data,
              batchCorrect = 'eb',
              powerTransformPhenotype = TRUE,
              removeLowVaryingGenes = 0.2,
              minNumSamples = 10,
              printOutput = TRUE,
              removeLowVaringGenesFrom = 'rawData')

if (!requireNamespace("BiocManager", quietly = TRUE))
    install.packages("BiocManager")
BiocManager::install("limma")
```

```
install.packages("ggplot2")
install.packages("ggpubr")

library(limma)
library(ggplot2)
library(ggpubr)

pFilter=0.001
risk=read.table(riskFile, header=T, sep="\t", check.names=F, row.names=1)
senstivity=read.csv(drugFile, header=T, sep=",", check.names=F, row.names=1)
colnames(senstivity)=gsub("(.*)\\_(\\d+)", "\\1", colnames(senstivity))

sameSample=intersect(row.names(risk), row.names(senstivity))
risk=risk[sameSample, "risk",drop=F]
senstivity=senstivity[sameSample,,drop=F]
rt=cbind(risk, senstivity)

rt$risk=factor(rt$risk, levels=c("low", "high"))
type=levels(factor(rt[,"risk"]))
comp=combn(type, 2)
my_comparisons=list()
for(i in 1:ncol(comp)){my_comparisons[[i]]<-comp[,i]}

for(drug in colnames(rt)[2:ncol(rt)]){
	rt1=rt[,c(drug, "risk")]
	colnames(rt1)=c("Drug", "Risk")
	rt1=na.omit(rt1)
	rt1$Drug=log2(rt1$Drug+1)
	test=wilcox.test(Drug ~ Risk, data=rt1)
	diffPvalue=test$p.value
	if(diffPvalue<pFilter){
		boxplot=ggboxplot(rt1, x="Risk", y="Drug", fill="Risk",
		                  xlab="Risk",
		                  ylab=paste0(drug, " senstivity"),
		                  legend.title="Risk",
		                  palette=c("#0066FF","#FF0000")
		                 )+
			stat_compare_means(comparisons=my_comparisons)
		pdf(file=paste0("drugSenstivity.", drug, ".pdf"), width=5, height=4.5)
		print(boxplot)
		dev.off()
	}
}
```
